# The ENK^RVM^ ^→SRD^ Projection Constitutes a Functionally Specific Circuit for Bidirectional Pain Modulation

**DOI:** 10.1002/cns.71064

**Published:** 2026-07-28

**Authors:** Kexing Wan, Jiajia Huang, Qian Xu, Chi Cui, Yulong Shi, Jie Lei, Zaiyan Fang, Tianyi Xu, Yufei Zheng, Jiaao Wu, Zhengle Zhu, Zhiqiang Zhang, Yujie Liang, Yichi Zhang, Hui‐Lin Pan, Ping Peng, Xianghong Jing, Man Li

**Affiliations:** ^1^ Hwamei College of Life and Health Sciences Zhejiang Wanli University Ningbo Zhejiang China; ^2^ School of Basic Medicine, Tongji Medical College, Key Laboratory of Neurological Diseases of Hubei Province and National Education Ministry Huazhong University of Science and Technology Wuhan China; ^3^ Clinical College of Chinese Medicine Hubei University of Chinese Medicine Wuhan China; ^4^ Department of Anesthesiology and Perioperative Medicine The University of Texas MD Anderson Cancer Center Houston Texas USA; ^5^ Department of Oncology, Tongji Hospital, Tongji Medical College Huazhong University of Science and Technology Wuhan Hubei China; ^6^ Institute of Acupuncture and Moxibustion China Academy of Chinese Medical Sciences Beijing China

**Keywords:** enkephalin, nucleus reticularis dorsalis, pain, rostral ventromedial medulla

## Abstract

**Aims:**

Chronic pain management remains a major clinical challenge due to the limited availability of effective therapies that avoid severe side effects. The rostral ventromedial medulla (RVM) is a critical brainstem center for pain modulation; however, the neurochemical identity and functional roles of its neuronal subpopulations remain incompletely understood.

**Methods:**

We performed a series of tests by constructing a complete Freund's adjuvant (CFA)‐induced inflammatory nociceptive model and a chronic constrictive injury (CCI) neuropathic pain model combined with behavioral experiments, in situ hybridization, chemogenetics, optogenetics, and in vivo electrophysiological techniques.

**Results:**

This study identifies enkephalinergic neurons in the RVM and their projections to the subnucleus reticularis dorsalis (SRD) as a distinct descending pain‐inhibitory circuit. RNAscope shows elevated ENK^RVM^ neuron activity in mouse models of inflammatory and neuropathic pain. Bidirectional chemogenetic and optogenetic manipulations demonstrate that inhibiting ENK^RVM^ neurons or their SRD projections induces hyperalgesia, while activation elevates baseline pain thresholds and reverses hyperalgesia in CFA and CCI models. This pathway modulates nociception selectively without affecting anxiety or motor function.

**Conclusion:**

These findings identify the ENK^RVM→SRD^ pathway as a bidirectional descending circuit for pain inhibition, highlighting its potential as a therapeutic target for analgesic intervention.

## Graphical abstract

Enkephalinergic neurons in the rostral ventromedial medulla form a functionally selective descending inhibitory microcircuit with the nucleus reticularis dorsalis that bidirectionally regulates nociception without engaging affective or motor components.

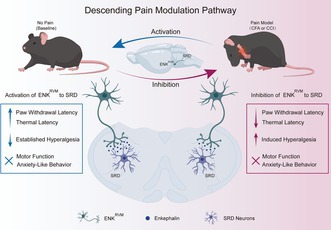

**Abbreviations**
AAVs: adeno‐associated viruses
ANOVA: analysis of variance
CCI: chronic constrictive injury
CFA: complete Freund's adjuvant
CPA: conditioned place aversion
CPP: conditioned place preference
ENK^RVM^ neurons: ENKergic neurons within the RVM
NA: numerical aperture
OD: outer diameter
RTPA: real‐Time Place Aversion
RTPP: real‐Time Place Preference
RVM: rostral ventromedial medulla
SRD: subnucleus reticularis dorsalis

## 1 Introduction

Pain, encompassing inflammatory and neuropathic conditions, represents a pervasive and debilitating health burden affecting a substantial proportion of the global population [1, 2]. The clinical management of pain remains a major challenge, as current first‐line therapies, particularly opioids, are frequently limited by inadequate efficacy, tolerance, and serious adverse effects, including addiction and respiratory depression [3, 4]. These limitations underscore the critical need to elucidate endogenous pain modulatory mechanisms in order to identify more precise and effective therapeutic targets.

Pain perception is not a passive reflection of peripheral noxious input but is actively shaped by descending modulatory pathways from the brain to the spinal cord. Among the core components of this descending system, the rostral ventromedial medulla (RVM) serves as a critical relay and integrative hub [5, 6]. Pioneering electrophysiological studies by Fields and colleagues established a functional framework for the RVM, defining three principal neuronal classes: ON‐cells that facilitate nociception, OFF‐cells that inhibit nociceptive transmission, and NEUTRAL‐cells with less well‐defined functions [7, 8]. The dynamic balance between ON‐ and OFF‐cell activity is thought to determine the net output of the RVM, thereby regulating the gain of spinal nociceptive transmission [9]. However, the neurochemical identity of these functionally defined neuronal populations, and how distinct neurochemical subsets contribute to different pain states, remains a long‐standing and unresolved question in the field [10].

Enkephalin (ENK), an endogenous opioid peptide, is abundantly expressed in the RVM [11]. ENK primarily acts on delta‐opioid receptors (DORs) and mu‐opioid receptors (MORs) to exert potent inhibitory effects on neuronal activity [12]. Early pharmacological studies suggested that ENK signaling within the RVM contributes to analgesia, potentially by inhibiting pain‐facilitatory neurons (such as ON‐cells) and/or disinhibiting pain‐inhibitory neurons [13, 14]. However, owing to the anatomical and neurochemical complexity of the RVM, the specific contribution of enkephalinergic RVM neurons (ENK^RVM^) as a distinct neuronal population remains poorly defined, including whether their activity is required for maintaining basal pain thresholds and whether they are engaged during chronic pain states to counteract hypersensitivity. Previous work has identified a descending RVM‐to‐spinal dorsal horn pathway composed largely of GABAergic/enkephalinergic neurons, which innervates the spinal dorsal horn and bidirectionally regulates nociceptive sensitivity [15]. This finding indicates that ENK‐expressing RVM neurons are not a homogeneous population, but may include anatomically and functionally distinct subpopulations with different downstream targets, including the spinal dorsal horn and brainstem reticular nuclei.

The RVM does not function in isolation but is embedded within a broader network of brainstem nuclei involved in pain processing. One key downstream target of the RVM is the nucleus reticularis dorsalis (SRD), located in the caudal medulla [16, 17, 18]. The SRD receives convergent nociceptive input, projects to thalamic regions, and is itself subject to descending control [19, 20]. Importantly, the RVM and SRD are reciprocally connected, forming a putative feedback loop that may be critical for fine‐tuning nociceptive processing [6, 21, 22]. Although the anatomical existence of the RVM–SRD pathway is well established, the functional role of specific neurochemical projections—particularly the enkephalinergic projection from the RVM to the SRD (ENK^RVM→SRD^)—remains largely unexplored. Dissecting this specific pathway may reveal whether pain modulation can be dissociated from other neurological functions, including affective and motivational processes.

In this study, we employed multidisciplinary approaches to define the role of ENK^RVM^ neurons and their projection to the SRD (ENK^RVM→SRD^) in pain modulation. We first assessed the activity of ENK^RVM^ neurons in mouse models of inflammatory and neuropathic pain. Using bidirectional chemogenetic and optogenetic manipulations in naïve mice, we then tested the necessity and sufficiency of this circuit in regulating basal nociception, as well as its involvement in affective and motor behaviors. We further examined whether activation of the ENK^RVM→SRD^ pathway could reverse established hypersensitivity in chronic pain models. Together, these approaches allowed us to determine whether the ENK^RVM→SRD^ projection constitutes a dedicated pain‐inhibitory circuit that is functionally dissociable from affective and motor side effects commonly associated with opioid analgesia. Our findings identify this pathway as a functionally selective descending system for pain modulation and provide new insights into the organization of brainstem pain control circuits.

## 2 Materials and Methods

### 2.1 Animals

Male mice (8–10 weeks old, 20–30 g) were randomly housed in groups of four per standard cage and provided with ad libitum access to food and water. Animals were maintained in a specific pathogen‐free (SPF) facility under a 12‐h light/dark cycle at 22°C–25°C with 40%–60% relative humidity. Wild‐type C57BL/6J mice were obtained from Beijing Witton Lihua Laboratory Animal Technology Co. Ltd. (Beijing, China). Penk‐ires‐cre mice (JAX:025112) were obtained from Shanghai Southern Model Biotechnology Co. Ltd. (Shanghai, China) and subsequently bred and maintained at Rat Noble Laboratory Animal Company (Wuhan, China). All experimental procedures were conducted in accordance with relevant guidelines and approved by the Institutional Animal Care and Use Committee (IACUC) of Huazhong University of Science and Technology.

### 2.2 Pain Model

#### 2.2.1 Inflammatory Pain Model (CFA)

Mice were anesthetized with 2.0% ~ 3.0% isoflurane delivered by inhalation. Chronic inflammatory pain was induced by intraplantar injection of 25 μL complete Freund's adjuvant (CFA) into the designated hind paw. Control mice received an equivalent volume of sterile saline.

#### 2.2.2 Neuropathic Pain Model (CCI)

Mice were anesthetized with 2.0% ~ 3.0% isoflurane. The skin overlying the left mid‐thigh was incised, and the biceps femoris muscle was bluntly separated to expose the sciatic nerve. Chronic constriction injury (CCI) was induced by placing three 4–0 chromic catgut ligatures around the sciatic nerve, spaced approximately 1 mm apart, until a slight constriction of the nerve was observed. Muscles were carefully repositioned, and the skin was sutured using absorbable sutures. Sham‐operated mice underwent the identical surgical procedure without nerve ligation, as previously described [23].

### 2.3 Behavioral Assessments

Behavioral assessments, including von Frey filament testing, the hot plate test, the open field test, conditioned place preference (CPP), conditioned place aversion (CPA), real‐time place aversion (RTPA), and real‐time place preference (RTPP), were performed as described in the Supporting Information S1.

### 2.4 Viral Microinjection and Optical Fiber Implantation

Mice were anesthetized with pentobarbital sodium (50 mg/kg, i.p.). The surgical field was prepared by shaving and disinfection with iodophor, and the eyes were protected with erythromycin ophthalmic ointment. Stereotaxic injections were performed using the following coordinates relative to bregma RVM; AP, −5.72 mm; ML, ±0.00 mm; DV, −5.70 mm and SRD; AP, −7.64 mm; ML, ±1.20 mm; DV, −5.00 mm. Viral vectors (Brinkes Biotechnology) were infused at a rate of 30 nL/min using a microinjector fitted with a glass micropipette (tip diameter 10–30 μm). After infusion, the micropipette was left in place for an additional 10 min to allow diffusion and prevent backflow. The scalp was sutured, and lidocaine/lincomycin ointment was applied locally for postoperative analgesia and infection prevention. Mice were allowed to recover on a heating pad. Behavioral testing was initiated 3 weeks after viral injection to ensure robust transgene expression.

### 2.5 Chemogenetics

For ENK^RVM^ manipulation, AAV2/9‐hSyn‐DIO‐mCherry, AAV2/9‐hSyn‐DIO‐hM4Di‐mCherry, AAV2/9‐hSyn‐DIO‐hM3Dq‐mCherry, or AAV9‐EF1α‐fDIO‐hM3Dq‐mCherry was injected into the RVM (80–100 nL). For ENK^RVM→SRD^ pathway manipulation, AAV9‐EF1α‐DIO‐retro‐FLEX‐FlpO was injected bilaterally into the SRD (80–100 nL per side). DREADD‐mediated activation or inhibition was achieved by intraperitoneal injection of clozapine‐N‐oxide (CNO; 3.0 mg/kg; working solution 0.3 mg/mL in saline; Enzo Life Sciences). Withdrawal thresholds were measured 1 h before and 1 h after CNO administration.

### 2.6 Optogenetics

For optogenetic manipulation, AAV2/9‐hSyn‐DIO‐mCherry, AAV2/9‐hSyn‐DIO‐eNpHR3.0‐mCherry, or AAV2/9‐hSyn‐DIO‐ChR2‐mCherry was injected into the RVM (80–100 nL). Two weeks after viral injection, an optical fiber (outer diameter, 200 μm; numerical aperture, 0.37; Inper) was implanted above the injection site. Behavioral testing was initiated 1 week after fiber implantation.

### 2.7 In Vivo Electrophysiology

AAV2/9‐hSyn‐DIO‐ChR2‐mCherry (100 nL) was injected into the RVM of Penk‐ires‐cre mice. Two weeks later, optoelectrodes were implanted in the target region. Neuronal activity recordings were performed 1 week after implantation.

### 2.8 In Vivo Electrophysiological Recordings

In vivo electrophysiological recordings were performed using the Zeus data acquisition system (Bio‐Signal Technologies, USA) in combination with NeuroExplorer 5 software (NEX Technologies, USA). Photoelectrodes consisting of 16 individually insulated nichrome wires (35 μm inner diameter; impedance 300–900 kΩ; Stablohm 675, California Filament Company) arranged in a 4 × 4 array with 200 μm spacing were used. An optical fiber was integrated at the center of the electrode array. The nucleus reticularis dorsalis (SRD; AP, −7.64 mm; ML, ±1.20 mm; DV, −5.00 mm, relative to bregma) was targeted for electrode implantation. The electrode assembly was secured using an 18‐pin connector (Mil‐Max) and fixed with dental acrylic (Shanghai New Century Company). Mice were allowed to recover for 1 week before recordings. Recordings were conducted in a cylindrical chamber lined with copper mesh, allowing free movement of the animals. Broadband neural signals (0.3 Hz–7.5 kHz) were continuously recorded at a sampling rate of 30 kHz using the Zeus system. Spike signals were extracted using a 300 Hz high‐pass filter. Real‐time spike sorting was performed using principal component analysis (PCA), followed by offline refinement using a Plexon spike‐sorting classifier.

### 2.9 RNAscope In Situ Hybridization

To assess the co‐localization of c‐Fos and ENK mRNA in the RVM, the RNAscope Multiplex Fluorescent Reagent Kit v2 (ACD Bio‐Techne) was used. Brain tissues were harvested 5 days after CFA injection and 11 days after CCI surgery. Mice were transcardially perfused with 25 mL phosphate‐buffered saline (PBS; Seville Bioscience), followed by 25 mL of 4% paraformaldehyde (PFA; Langercoscience). Brains were post‐fixed overnight in 4% PFA, rinsed in PBS, and cryoprotected by sequential immersion in 20% and 30% sucrose solutions. Tissues were embedded in optimal cutting temperature (OCT) compound (Tissue‐Tek), cryosectioned at 14 μm, mounted on Superfrost Plus adhesive slides, and stored at −80°C until use. RNAscope in situ hybridization was performed according to the manufacturer's instructions using probes targeting mouse c‐Fos (catalog no. 316921) and mouse ENK (catalog no. 318761).

### 2.10 Image Acquisition and Quantification

Tissue sections were imaged at 10× magnification using a fully automated SLIDEVIEW VS200 system (Olympus). c‐Fos– and enkephalin (ENK)‐positive neurons were manually counted using ImageJ software (NIH). Quantitative analyses included 3–4 mice per group. Marker expression was quantified by calculating the percentage of marker‐positive cells per mouse, followed by averaging these values across animals within each experimental group.

### 2.11 Statistical Analysis

The investigator responsible for data analysis was blinded to group allocation. Statistical analyses were performed using GraphPad Prism version 9.0. For data that did not follow a normal distribution, group differences were assessed using the Kruskal–Wallis test followed by Dunn's post hoc test. For normally distributed data, two‐tailed unpaired *t*‐tests and one‐way or two‐way analysis of variance (ANOVA) were applied, followed by Tukey's or Bonferroni's post hoc tests as appropriate. Data are presented as the mean ± standard error of the mean (SEM). Differences were considered statistically significant at *p* < 0.05.

## 3 Results

### 3.1 ENK^RVM^ Neurons Are Significantly Activated Under Pain‐Hypersensitive Conditions

**FIGURE 1:**
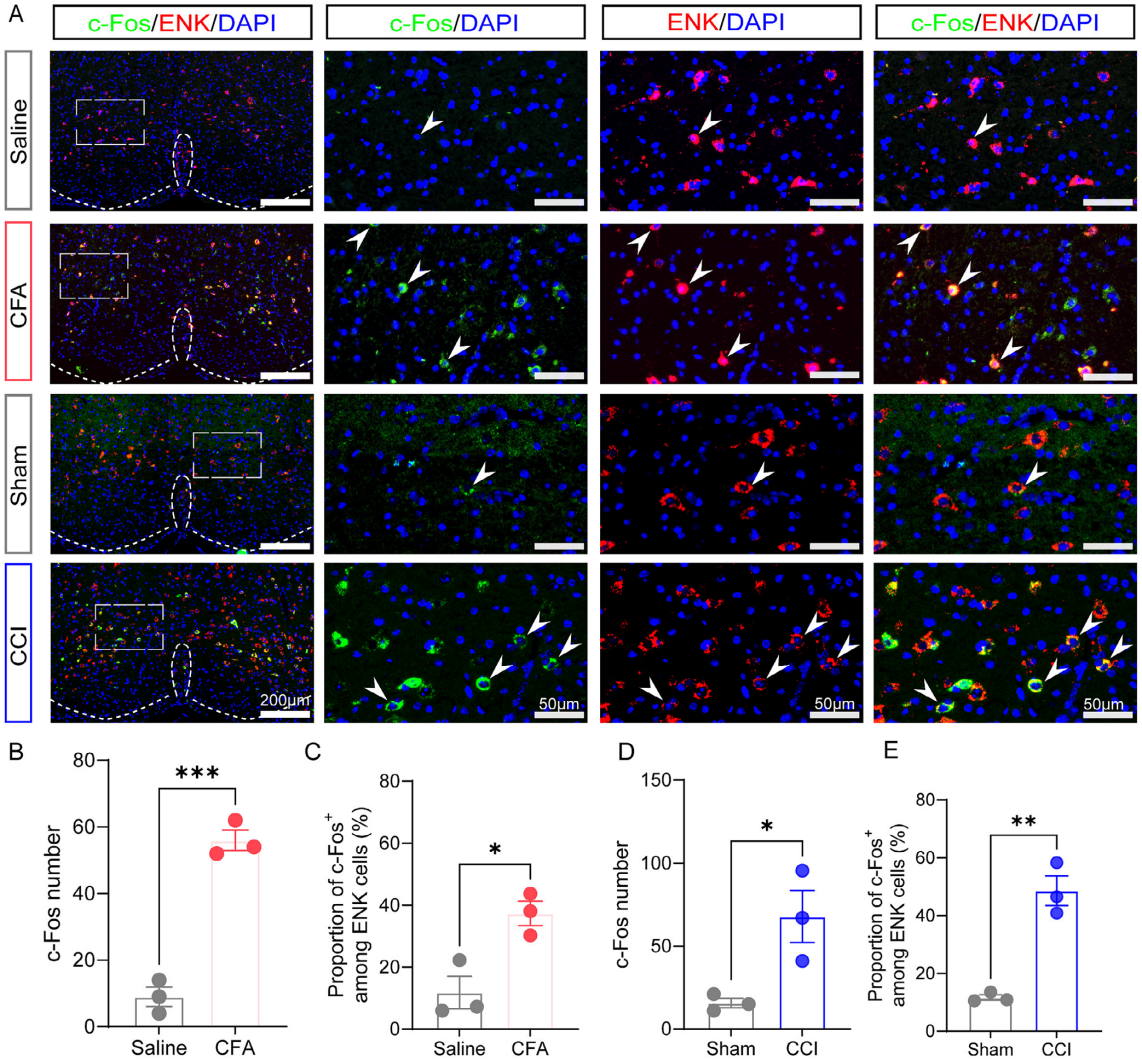
ENK^RVM^ neuronal activity is enhanced in inflammatory and neuropathic pain models. (A) Representative RNAscope in situ hybridization images showing enkephalin (ENK, red) and c‐Fos (green) expression in the RVM. Scale bars = 200 μm. Right panels show 4× magnified views of the boxed regions in the left panels. Scale bars = 50 μm. (B) Quantification of c‐Fos–positive cells in the RVM of saline‐ and CFA‐treated mice. (C) Proportion of ENK‐positive neurons co‐expressing c‐Fos in the RVM of saline‐ and CFA‐treated mice. (D) Quantification of c‐Fos–positive cells in the RVM of Sham‐ and CCI‐treated mice. (E) Proportion of ENK‐positive neurons co‐expressing c‐Fos in the RVM of Sham‐ and CCI‐treated mice. **p < 0.01, saline versus CFA or sham versus CCI; two‐tailed unpaired *t*‐test. For RNAscope quantification, *n* = 3 mice per group. All data are shown as mean ± SEM.

To determine the activity of ENK‐positive RVM neurons associated with pain hypersensitivity, we first established complete Freund's adjuvant (CFA)‐induced inflammatory pain and chronic constriction injury (CCI)‐induced neuropathic pain models. Behavioral testing confirmed that both models robustly induced persistent mechanical and thermal hyperalgesia (Figure S1A–D). RNAscope in situ hybridization revealed a significant increase in c‐Fos–positive cells within the RVM of both pain model groups compared with controls (Figure 1A,B,D). Further colocalization analysis demonstrated a marked increase in the number of neurons co‐labeled for c‐Fos and ENK (Figure 1C,E). These findings indicate that ENK^RVM^ neuronal activity is significantly enhanced under both inflammatory and neuropathic pain conditions.

### 3.2 Inhibition of ENK^RVM^ Neurons Induces Mechanical and Thermal Hypersensitivity and Aversive Behavior

**FIGURE 2:**
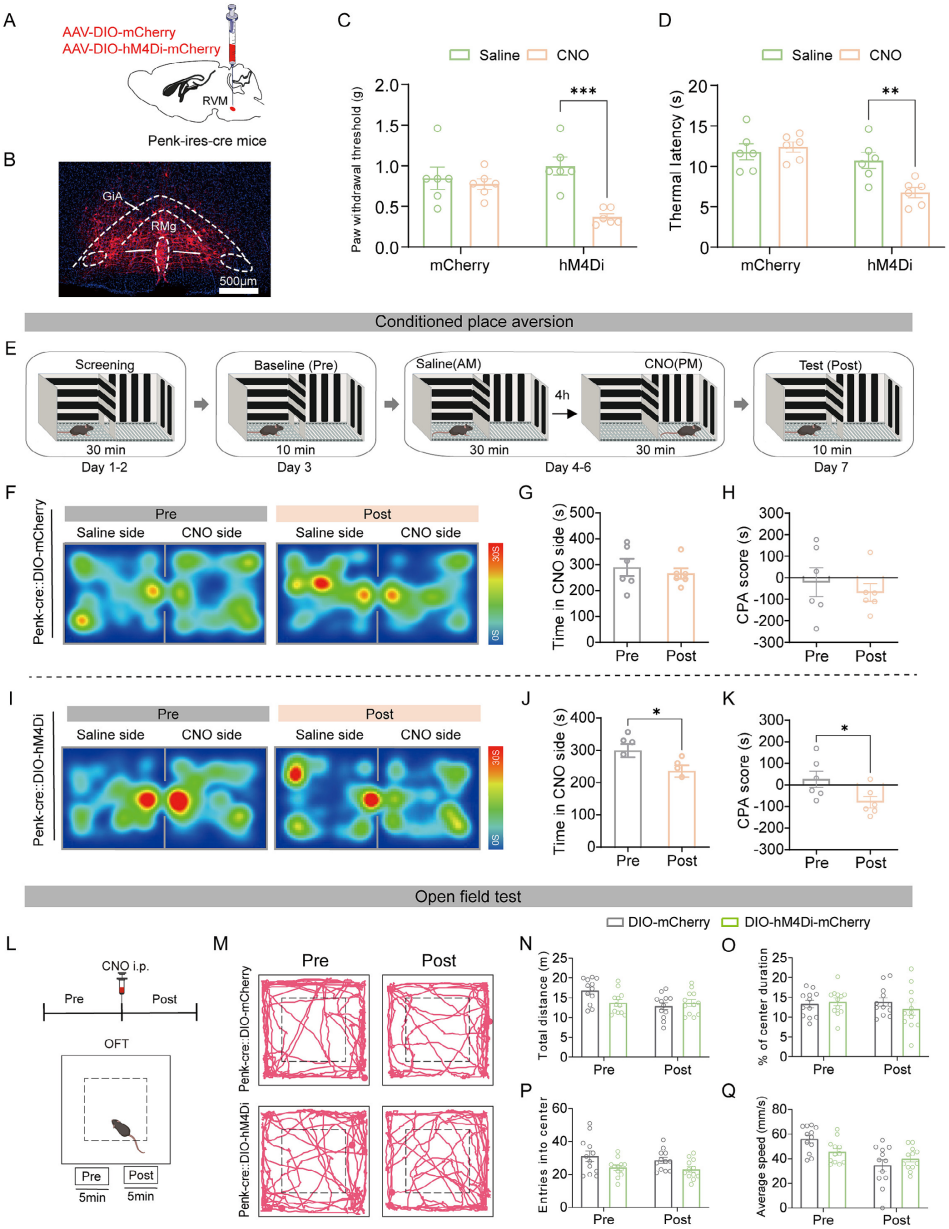
Chemogenetic inhibition of ENK^RVM^ neurons induces hyperalgesia and place avoidance behavior without affecting locomotion. (A) Schematic illustration of stereotaxic injection of AAV‐DIO‐hM4Di‐mCherry or control AAV‐DIO‐mCherry into the RVM of Penk‐ires‐cre mice. (B) Representative fluorescence image showing viral expression within the RVM. Scale bar, 500 μm. (C, D) Chemogenetic inhibition of ENK^RVM^ neurons significantly reduced mechanical paw withdrawal thresholds (C) and thermal withdrawal latencies (D) following CNO administration. *n* = 6 mice per group. **p < 0.01, ***p < 0.001 versus saline treatment within the same viral group; two‐way ANOVA followed by Bonferroni's post hoc test. (E) Experimental design of the conditioned place aversion (CPA) paradigm. (F) Representative trajectory maps of Penk‐cre::DIO‐mCherry mice during CPA testing before and after saline or CNO conditioning. (G, H) Time spent in the CNO‐paired chamber (G) and CPA score (H) in Penk‐cre::DIO‐mCherry control mice. (I) Representative trajectory maps of Penk‐cre::DIO‐hM4Di mice during CPA testing before and after saline or CNO conditioning. (J, K) Chemogenetic inhibition of ENK^RVM^ neurons induced significant place aversion, as reflected by reduced time spent in the CNO‐paired chamber (J) and decreased CPA scores (K). *n* = 6 mice per group. *p < 0.05. (L) Schematic of the open field test (OFT). (M) Representative locomotor trajectories of Penk‐cre::DIO‐mCherry and Penk‐cre::DIO‐hM4Di mice before and after CNO injection. (N–Q) Chemogenetic inhibition of ENK^RVM^ neurons did not alter total distance traveled (N), time spent in the center (O), center entries (P), or average speed (Q) in the OFT. *n* = 12 mice per group. All data are presented as mean ± SEM.

To determine whether ENK^RVM^ neurons are required for maintaining basal nociceptive thresholds, AAV‐DIO‐hM4Di‐mCherry was injected into the RVM to selectively inhibit ENK^RVM^ neurons in naïve Penk‐ires‐cre mice (Figure 2A,B). Behavioral analyses revealed that chemogenetic inhibition of ENK^RVM^ neurons resulted in a significant reduction in mechanical and thermal withdrawal thresholds compared with control mice (Figure 2C,D), indicating that ENK^RVM^ neuronal inhibition is sufficient to induce mechanical and thermal hyperalgesia.

We next examined whether inhibition of ENK^RVM^ neurons elicits negative affective responses using a conditioned place aversion (CPA) paradigm (Figure 2E). Penk‐ires‐cre mice expressing hM4Di displayed a significant reduction in time spent in the CNO‐paired chamber and a corresponding decrease in CPA scores compared with Penk‐ires‐cre mice expressing control DIO‐mCherry (Figure 2F–K), However, direct comparison of CPA scores between mCherry control and hM4Di‐expressing mice did not reach statistical significance (Figure S2).

To exclude the possibility that the observed hyperalgesia resulted from altered motor performance or anxiety, locomotor activity was assessed using the open‐field test (Figure 2L). Baseline activity was recorded prior to CNO administration, followed by post‐inhibition measurements 1 h after injection (Figure 2M). Chemogenetic inhibition of ENK^RVM^ neurons did not alter total distance traveled, time spent in the center, center entries, or average velocity (Figure 2N–Q), indicating that motor function and anxiety‐like behavior were unaffected.

We further validated these findings using optogenetic inhibition. Injection of AAV‐DIO‐eNpHR3.0‐mCherry into the RVM of Penk‐ires‐cre mice enabled light‐mediated inhibition of ENK^RVM^ neurons (Figure S3A,B). Optical inhibition (589 nm, 1 Hz, 10 mW) produced a rapid and reversible reduction in mechanical and thermal withdrawal thresholds, with recovery following cessation of stimulation (Figure S3C,D). In real‐time place avoidance assays, optogenetic inhibition induced immediate avoidance of the stimulated compartment, which returned to baseline within 10 min after light offset (Figure S3E,H,I). No avoidance behavior was observed in DIO‐mCherry control mice (Figure S3F,G). Open‐field testing confirmed that optogenetic inhibition did not affect locomotor activity or baseline anxiety levels (Figure S3J–M). Together, these results demonstrate that inhibition of ENK^RVM^ neurons is sufficient to induce mechanical and thermal hyperalgesia without affecting motor performance or anxiety‐like behavior. The CPA data suggest a potential aversive effect, whereas the optogenetic RTPA experiment provides stronger time‐locked evidence that ENK^RVM^ neuronal inhibition induces avoidance behavior.

### 3.3 Activation of ENK^RVM^ Neurons Increases Basal Pain Thresholds

**FIGURE 3:**
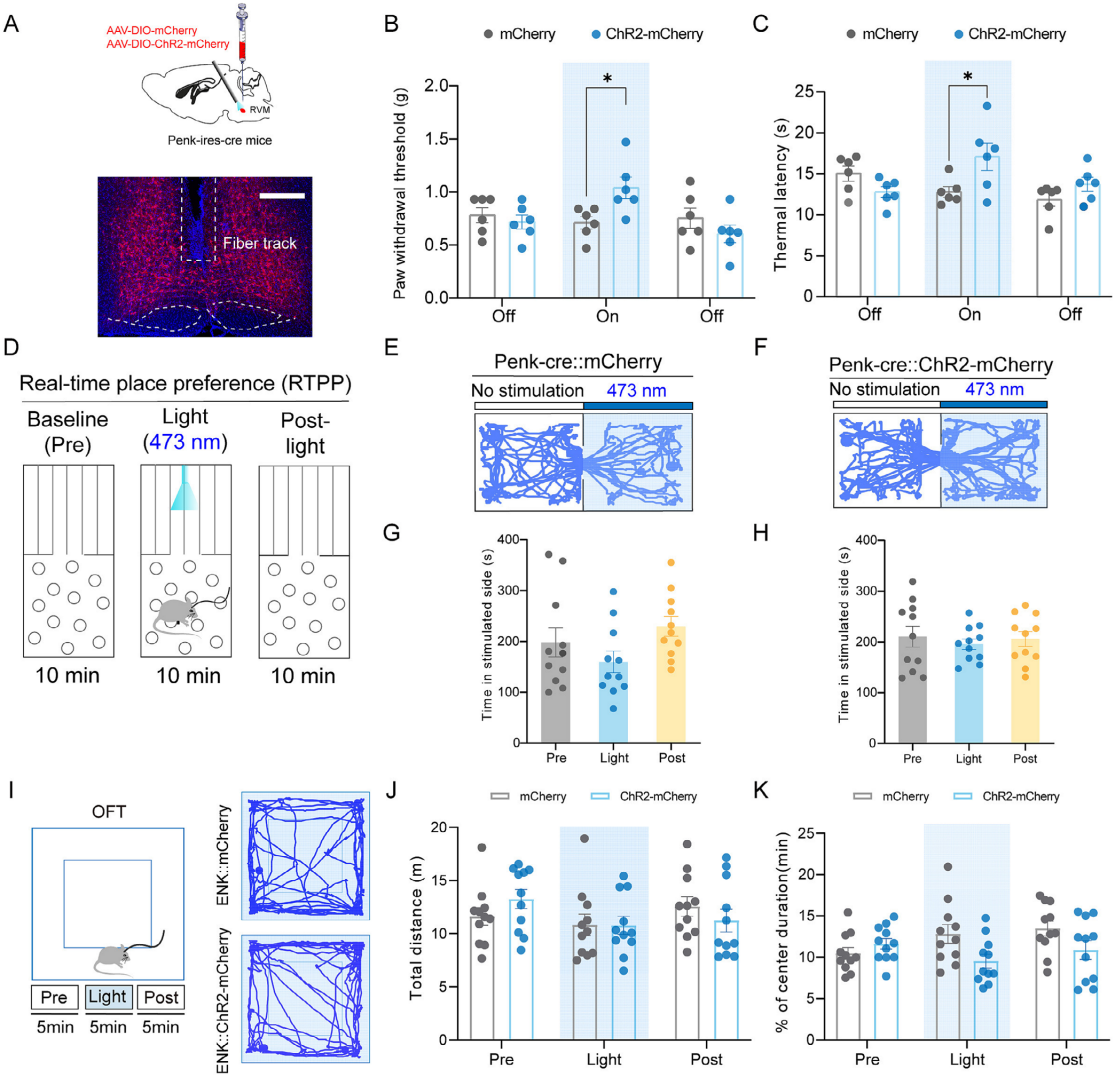
Optogenetic activation of ENK^RVM^ elevates basal pain thresholds without inducing reward or locomotor alterations. (A) Schematic illustrating viral delivery and optical stimulation strategy for expressing AAV‐DIO‐ChR2‐mCherry or control AAV‐DIO‐mCherry in the RVM of Penk‐ires‐cre mice. Bottom: Representative fluorescence image showing viral expression and fiber track within the RVM. Scale bar, 500 μm (B, C) Blue light stimulation (473 nm, 20 Hz, 5‐ms pulse width, ~5 mW) of the RVM significantly increased mechanical paw withdrawal thresholds (B) and thermal withdrawal latencies in the hot plate test (C) in mice expressing ChR2, but not in control mice expressing mCherry. *n* = 6 mice per group. (D) Schematic of the real‐time place preference (RTPP) paradigm. (E, F) Representative trajectory maps during RTPP testing in Penk‐ires‐cre mice injected with AAV‐DIO‐mCherry (E) or AAV‐DIO‐ChR2‐mCherry (F) in the RVM. (G, H) Quantification of time spent in the light‐paired chamber before (Pre), during (Light), and after (Post) a 10‐min optical stimulation session in control mice (G) and ChR2‐expressing mice (H), showing no significant place preference induced by ENK^RVM^ activation. *n* = 12 mice per group. (I) Schematic of the open field test (OFT). (J, K) Quantification of total distance traveled (J) and proportion of time spent in the center versus periphery (K) in the OFT before (Pre), during (Light), and after (Post) 5 min of optical stimulation, indicating no significant effects on locomotor activity or anxiety‐like behavior, *n* = 12 mice per group. All data are presented as mean ± SEM. *p < 0.05.

To examine whether ENK^RVM^ neuronal activation is sufficient to elevate basal nociceptive thresholds, optogenetic activation experiments were performed in naive, pain‐free Penk‐ires‐cre mice by injecting AAV‐DIO‐ChR2‐mCherry into the RVM (Figure 3A). Behavioral analyses revealed that blue light stimulation of ENK^RVM^ neurons significantly elevated mechanical and thermal withdrawal thresholds in naïve mice, with a time‐locked recovery following cessation of stimulation (Figure 3B,C), demonstrating that activation of ENK^RVM^ neurons elevates basal pain thresholds.

In the same cohort, real‐time place preference (RTPP) testing showed that optical activation of ENK^RVM^ neurons did not alter the time spent on the stimulated side (Figure 3D–H). Consistently, open‐field testing revealed no significant changes in total locomotor distance or the proportion of time spent in the central zone (Figure 3I–K), indicating that ENK^RVM^ activation does not induce reward‐related or anxiety‐like behavioral changes.

These findings were independently validated using a chemogenetic approach. Following injection of AAV‐DIO‐hM3Dq‐mCherry into the RVM of Penk‐ires‐cre mice, chemogenetic activation of ENK^RVM^ neurons similarly elevated mechanical and thermal withdrawal thresholds (Figure S4A–D). Conditioned place preference (CPP) testing revealed no significant differences in chamber occupancy time or CPP scores between Penk‐ires‐cre::DIO‐hM3Dq mice and control Penk‐ires‐cre::DIO‐mCherry mice after CNO pairing (Figure S4E–K). Open‐field analysis further confirmed that chemogenetic activation did not affect locomotor activity or anxiety‐like behavior (Figure S4L–Q). Collectively, these data demonstrate that activation of ENK^RVM^ neurons selectively elevates basal nociceptive thresholds without altering locomotor function, reward processing, or anxiety‐related behaviors.

### 3.4 Activation of ENK^RVM^ Neurons Alleviates Inflammatory and Neuropathic Pain Hypersensitivity

**FIGURE 4:**
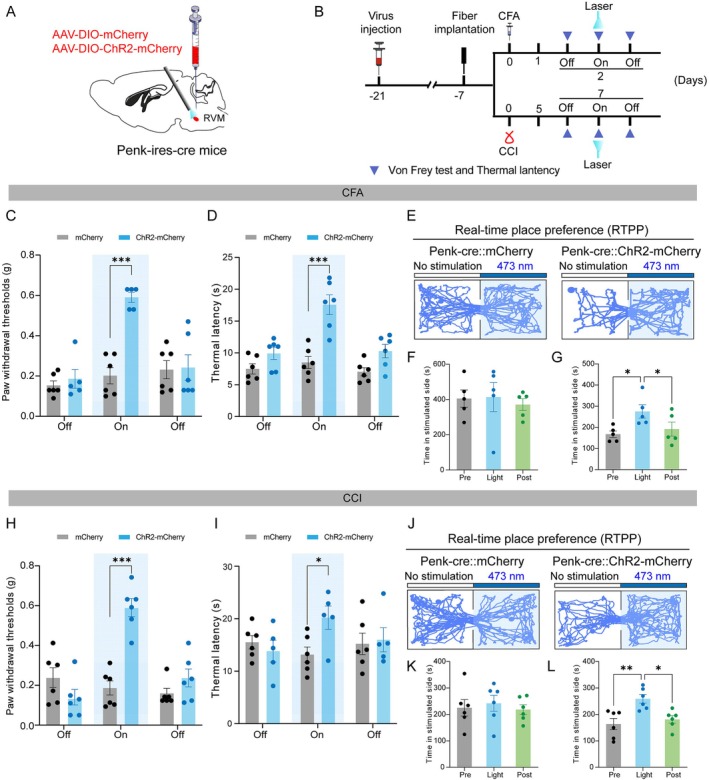
Optogenetic activation of ENK^RVM^ neurons reverses inflammatory and neuropathic pain hypersensitivity. (A) Schematic illustrating stereotaxic viral injection for the expression of AAV‐DIO‐ChR2‐mCherry or control AAV‐DIO‐mCherry in the RVM of Penk‐ires‐cre mice. (B) Experimental timeline and behavioral testing paradigm. (C, D) In CFA‐induced inflammatory pain mice, blue light stimulation of the RVM (473 nm, 20 Hz, 5‐ms pulse width, ~5 mW) significantly increased mechanical paw withdrawal thresholds (C) and thermal withdrawal latencies in the hot plate test (D) in ChR2‐expressing mice, but not in control mice, *n* = 6 mice per group. (E) Representative real‐time place preference (RTPP) trajectories from CFA‐treated mice injected with AAV‐DIO‐mCherry (left) or AAV‐DIO‐ChR2‐mCherry (right) in the RVM. (F, G) Quantification of time spent in the light‐paired chamber before (Pre), during (Light), and after (Post) a 10‐min optical stimulation session in CFA‐treated control mice (F) and ChR2‐expressing mice (G), demonstrating a significant stimulation‐induced place preference in the ChR2 group. (H, I) In CCI‐induced neuropathic pain mice, optogenetic activation of ENK^RVM^ neurons significantly increased mechanical paw withdrawal thresholds, *n* = 5 mice per group. (H) and thermal withdrawal latencies in the hot plate test (I) in ChR2‐expressing mice, but not in control mice. (J) Representative RTPP trajectories from CCI‐treated mice injected with AAV‐DIO‐mCherry (left) or AAV‐DIO‐ChR2‐mCherry (right) in the RVM. (K, L) Quantification of time spent in the light‐paired chamber before (Pre), during (Light), and after (Post) optical stimulation in CCI‐treated control mice (K) and ChR2‐expressing mice (L), indicating that activation of ENK^RVM^ neurons produces a robust place preference in neuropathic pain conditions, *n* = 6 mice per group. All data are presented as mean ± SEM. *p < 0.05, **p < 0.01, ***p < 0.001.

To determine whether ENK^RVM^ neuronal activation alleviates inflammatory and neuropathic pain, AAV‐DIO‐ChR2‐mCherry or control AAV‐DIO‐mCherry was injected into the RVM of Penk‐ires‐cre mice. After 21 days of viral expression, baseline nociceptive thresholds were assessed, followed by induction of CFA‐induced inflammatory pain or CCI‐induced neuropathic pain (Figure 4A,B). Behavioral analyses revealed that optogenetic activation of ENK^RVM^ neurons significantly elevated mechanical and thermal withdrawal thresholds in both CFA and CCI models (Figure 4C,D,H,I). Real‐time place preference (RTPP) testing further revealed that optical stimulation increased the time spent on the stimulated side in both pain models (Figure 4E–G,J–L), consistent with relief from ongoing pain. These findings were independently validated using a chemogenetic approach (Figure S5A–C,H–J). Following successful establishment of pain models, systemic administration of CNO to activate ENK^RVM^ neurons similarly elevated mechanical and thermal withdrawal thresholds in both CFA‐ and CCI‐treated mice (Figure S5D,E,K,L). Notably, these effects returned to baseline approximately 2.5 h after CNO administration (Figure S5F,G,M,N). Collectively, these data indicate that activation of ENK^RVM^ neurons effectively alleviates inflammatory and neuropathic pain hypersensitivity.

### 3.5 Characterization of the ENK^RVM^ ^→SRD^ Projection Reveals a Circuit That Bidirectionally Modulates Basal Pain Thresholds

**FIGURE 5:**
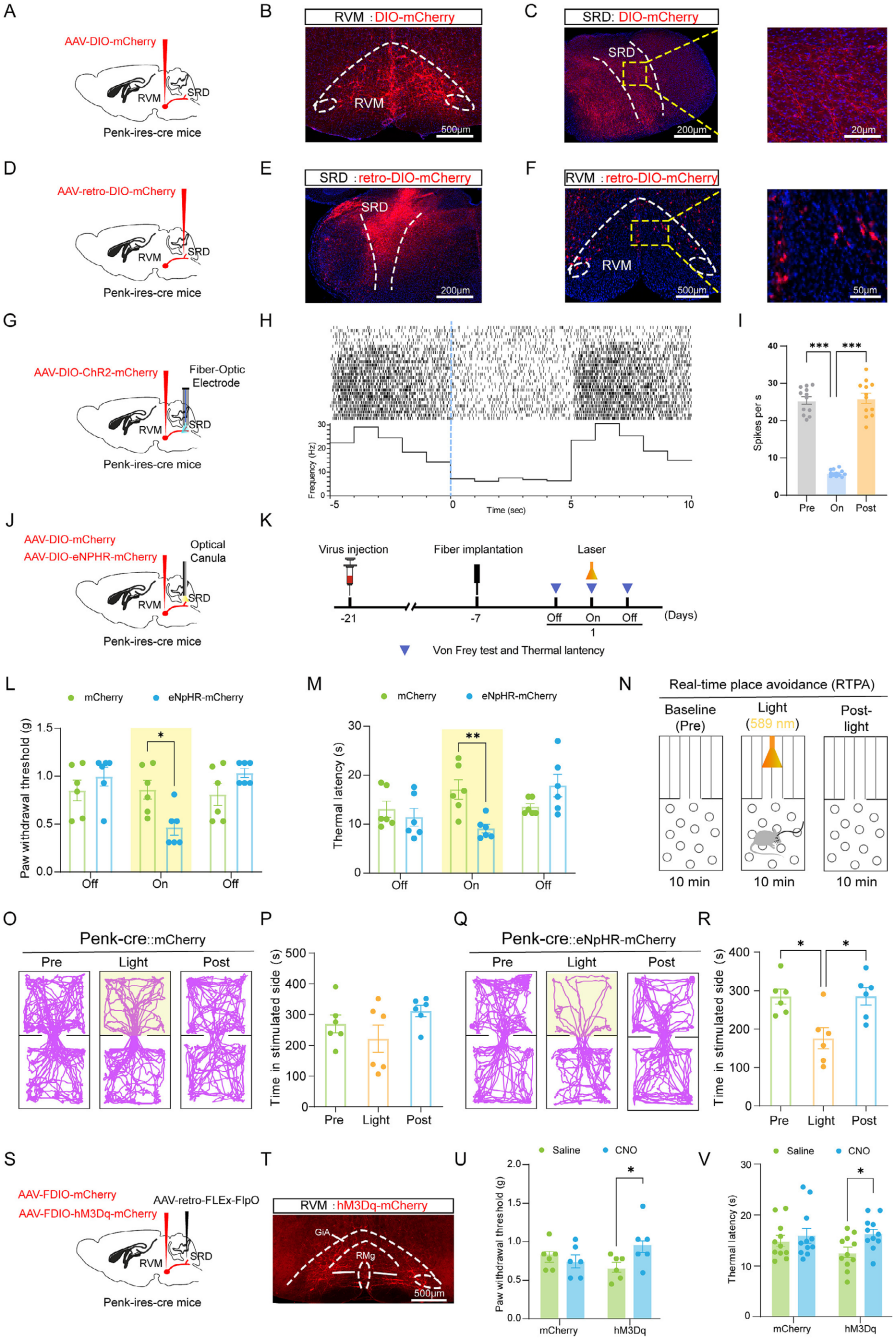
Characterization of the ENK^RVM→SRD^ projection reveals a circuit that bidirectionally modulates basal nociception and aversive behavior. (A) Schematic illustrating the viral anterograde tracing strategy used to label projections from enkephalinergic RVM neurons. (B) Representative fluorescence image of the RVM following anterograde viral labeling. Scale bar = 500 μm. (C) Representative fluorescence image of the nucleus reticularis dorsalis (SRD) showing dense ENK^RVM^ axonal projections. Scale bar = 200 μm. Right panel shows a 10× magnified view of the region outlined by the dashed yellow box. (D) Schematic illustrating the viral retrograde tracing strategy from the SRD. (E) Representative fluorescence image of the SRD at the injection site of the retrograde tracer. Scale bar = 200 μm. (F) Representative fluorescence image of the RVM showing retrogradely labeled ENK‐expressing neurons projecting to the SRD. Scale bar = 500 μm. Right panel shows an 8× magnified view of the region outlined by the dashed yellow box. (G) Schematic of optogenetic stimulation combined with in vivo electrophysiological recording to assess functional connectivity between ENK^RVM^ terminals and SRD neurons. (H) Representative raster plot (top) and peri‐stimulus time histogram (PSTH; bottom) illustrating SRD neuronal firing aligned to optical activation of ENK^RVM^ axon terminals. (I) Quantification of SRD neuronal firing rates before, during, and after optical stimulation, demonstrating a significant suppression of SRD neuronal activity upon ENK^RVM^ terminal activation (For in vivo optoelectrophysiological recordings, *n* = 12 neurons from 3 mice). (J) Schematic illustrating stereotaxic viral injection for optogenetic inhibition of ENK^RVM→SRD^ neurons via expression of AAV‐DIO‐eNpHR3.0‐mCherry or control AAV‐DIO‐mCherry in the RVM of Penk‐ires‐cre mice, with optical fibers implanted above the SRD. (K) Experimental timeline and behavioral testing paradigm. (L, M) Yellow light illumination (589 nm, 1 Hz, 999‐ms pulse width, 10 mW) of the SRD significantly decreased mechanical paw withdrawal thresholds (L) and thermal withdrawal latencies (M) in mice expressing eNpHR3.0, but not in control mice expressing mCherry, indicating that inhibition of ENK^RVM→SRD^ neurons induces mechanical and thermal hyperalgesia. (N) Schematic of the real‐time place avoidance (RTPA) paradigm. (O,P) Representative trajectory maps (O) and quantification (P) of RTPA behavior before (Pre), during (Light), and after (Post) a 10‐min yellow light stimulation session in control mice expressing AAV‐DIO‐mCherry, showing no significant place avoidance. (Q, R) Representative trajectory maps (Q) and quantification (R) of RTPA behavior in mice expressing AAV‐DIO‐eNpHR3.0‐mCherry, demonstrating robust light‐induced place avoidance during ENK^RVM→SRD^ neuronal inhibition. (S) Schematic illustrating an intersectional chemogenetic strategy for selective activation of the ENK^RVM→SRD^ projection via injection of AAV‐FDIO‐hM3Dq‐mCherry or control AAV‐FDIO‐mCherry into the RVM and AAV‐retro‐FLEx‐FlpO into SRD. (T) Representative fluorescence image confirming viral expression at the injection sites. Scale bar = 500 μm. (U, V) Chemogenetic activation of the ENK^RVM→SRD^ pathway significantly increased mechanical paw withdrawal thresholds (U) and thermal withdrawal latencies (V), indicating enhanced basal antinociception, *n* = 6 mice per group. All data are presented as mean ± SEM. Statistical significance was assessed as indicated: *p < 0.05, **p < 0.01 and ***p < 0.001.

We next investigated the anatomical projections of enkephalinergic RVM neurons. An anterograde viral tracer was injected into the RVM of Penk‐ires‐cre mice (Figure 5A). After validating the injection site, abundant labeled axonal fibers were observed in the SRD using whole‐brain imaging (Figure 5B,C), indicating that ENK‐expressing RVM neurons project to the SRD. To further confirm this projection, a retrograde viral tracer was injected into the SRD of Penk‐ires‐cre mice (Figure 5D). Following verification of the injection site, retrogradely labeled neuronal cell bodies were detected in the RVM (Figure 5E,F). SRD‐projecting ENK^RVM^ neurons accounted for 35.06% of the total ENK‐positive neuronal population in the RVM, indicating that the ENK^RVM→SRD^ pathway represents a distinct subpopulation of RVM enkephalinergic neurons (Figure S6A,B). Further confirming the RVM‐to‐SRD projection of ENK‐expressing neurons. We next examined the functional connectivity of this projection using in vivo optoelectrophysiology. In Penk‐ires‐cre mice, AAV‐DIO‐ChR2‐mCherry was injected into the RVM, and an optoelectrode was implanted in the SRD (Figure 5G). Twenty‐one days after viral expression, blue light was delivered to activate ENK‐expressing RVM axon terminals while simultaneously recording the firing activity of SRD neurons. Optical activation significantly reduced the firing frequency of SRD neuronal cell bodies (Figure 5H,I), indicating that enkephalinergic RVM neurons form a functional inhibitory projection to the SRD.

The RVM and SRD are reciprocally connected, forming a key node in descending pain modulation [24]. We therefore examined whether selective manipulation of the ENK^RVM→SRD^ projection regulates basal nociceptive sensitivity. To inhibit this pathway, AAV‐DIO‐eNpHR3.0‐mCherry was injected into the RVM and an optical cannula was implanted into the SRD of Penk‐ires‐cre mice, enabling optogenetic inhibition of ENK^RVM^ axon terminals in the SRD (Figure 5J,K). Behavioral analyses revealed that optogenetic inhibition of the ENK^RVM→SRD^ projection in naïve mice significantly reduced mechanical and thermal withdrawal thresholds (Figure 5L,M). In parallel, inhibition of this pathway decreased the time spent in the light‐paired compartment during real‐time place aversion testing (Figure 5N–R), indicating that suppression of ENK^RVM→SRD^ activity induces aversive motivational behavior. To determine whether activation of this projection exerts opposite effects, ENK^RVM→SRD^ neurons were selectively activated using an intersectional chemogenetic strategy (Figure 5S,T). Specifically, AAV‐FDIO‐hM3Dq‐mCherry was injected into the RVM and AAV‐retro‐FLEx‐FlpO was injected into the SRD of Penk‐ires‐cre mice, enabling chemogenetic activation of ENK^RVM^ neurons projecting to the SRD. Activation of the ENK^RVM→SRD^ projection significantly increased mechanical and thermal withdrawal thresholds in naïve mice (Figure 5U,V). Because ENK‐expressing RVM neurons projecting to the spinal dorsal horn have been reported previously, to evaluate the projection specificity of this intersectional viral strategy, we also examined spinal cord sections from the same animals. Although robust viral labeling was observed in the RVM, no obvious labeled somata or axonal projections were detected in the spinal dorsal horn (Figure S7A,B). Thus, within the detection limit of our histological analysis, this strategy preferentially labeled the ENK^RVM→SRD^ pathway and did not produce detectable labeling of direct RVM‐to‐spinal dorsal horn projections.

Importantly, neither optogenetic inhibition nor chemogenetic activation of the ENK^RVM→SRD^ circuit affected average locomotor speed, total distance traveled, time spent in the central area, or center‐entry frequency in the open‐field test (Figure S8A–J), indicating that modulation of this pathway does not alter baseline locomotor activity or anxiety‐like behavior.

Together, these data demonstrate that the ENK^RVM→SRD^ projection bidirectionally regulates basal nociceptive sensitivity while remaining functionally dissociable from locomotor and anxiety‐related behaviors.

### 3.6 Activation of the ENK^RVM^ ^→SRD^ Projection Alleviates Inflammatory and Neuropathic Pain Hypersensitivity

**FIGURE 6:**
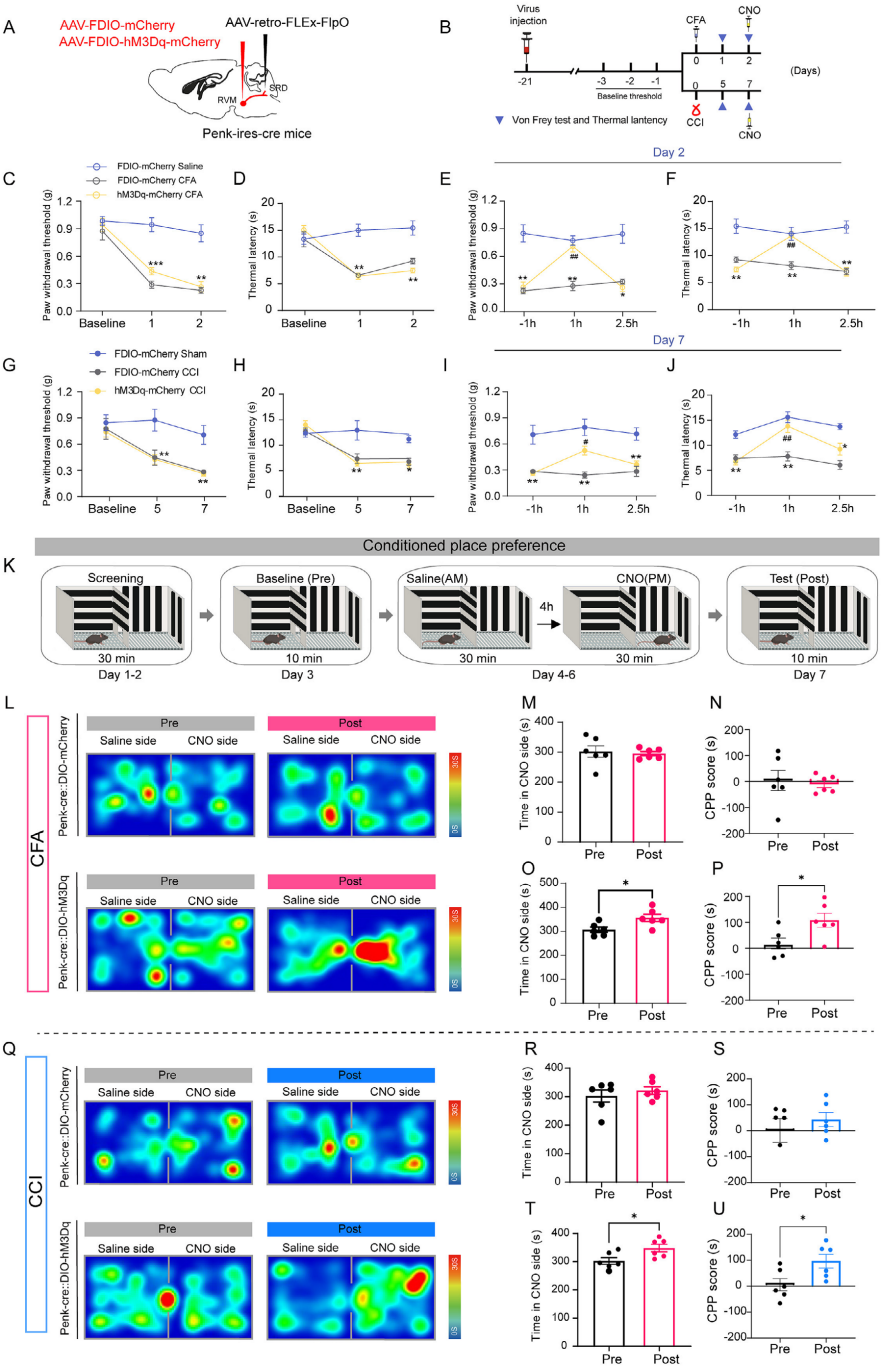
Chemogenetic activation of the ENK^RVM→SRD^ pathway reverses inflammatory and neuropathic pain hypersensitivity and produces relief‐associated reward. (A) Schematic illustrating an intersectional chemogenetic strategy for selective activation of the ENK^RVM→SRD^ projection via injection of AAV‐FDIO‐hM3Dq‐mCherry or control AAV‐FDIO‐mCherry into the RVM and AAV‐retro‐FLEx‐FlpO into the SRD of Penk‐ires‐cre mice. (B) Experimental timeline and behavioral testing paradigm for chemogenetic manipulation of the ENK^RVM→SRD^ pathway in CFA‐ and CCI‐induced pain models. (C, D) Time course of mechanical paw withdrawal thresholds (C) and thermal withdrawal latencies (D) in the CFA‐induced inflammatory pain model. (E, F) Chemogenetic activation of the ENK^RVM→SRD^ pathway significantly increased mechanical paw withdrawal thresholds (E) and thermal withdrawal latencies (F) in CFA‐treated mice following CNO administration, with effects returning to baseline approximately 2.5 h after injection. (G, H) Time course of mechanical paw withdrawal thresholds (G) and thermal withdrawal latencies (H) in the CCI‐induced neuropathic pain model. (I, J) Chemogenetic activation of the ENK^RVM→SRD^ pathway significantly increased mechanical paw withdrawal thresholds (I) and thermal withdrawal latencies (J) in CCI‐treated mice following CNO administration, with effects returning to baseline approximately 2.5 h after injection. **p < 0.01, ***p < 0.001 versus saline or sham group; #*p* < 0.05, ##*p* < 0.01 versus CFA‐ or CCI‐treated control group. Statistical significance was determined by two‐way ANOVA followed by Bonferroni post hoc tests. (K) Schematic of the conditioned place preference (CPP) paradigm. (L) Representative CPP trajectories of CFA‐treated mice before and after saline‐ or CNO‐conditioning. (M, N) Time spent in the CNO‐paired chamber (M) and CPP score (N) in CFA‐treated control mice expressing AAV‐FDIO‐mCherry. (O, P) Time spent in the CNO‐paired chamber (O) and CPP score (P) in CFA‐treated mice expressing AAV‐FDIO‐hM3Dq‐mCherry, demonstrating significant relief‐associated place preference. (Q) Representative CPP trajectories of CCI‐treated mice before and after saline‐ or CNO‐conditioning. (R, S) Time spent in the CNO‐paired chamber (R) and CPP score (S) in CCI‐treated control mice. (T, U) Time spent in the CNO‐paired chamber (T) and CPP score (U) in CCI‐treated mice expressing AAV‐FDIO‐hM3Dq‐mCherry, indicating robust preference associated with pain relief. *n* = 6 mice per group. Data are presented as mean ± SEM.

We next examined whether activation of the ENK^RVM→SRD^ projection alleviates inflammatory and neuropathic pain hypersensitivity. Penk‐ires‐cre mice received injections of AAV‐FDIO‐hM3Dq‐mCherry into the RVM and AAV‐retro‐FLEx‐FlpO into the SRD. After 21 days of viral expression, baseline mechanical nociceptive thresholds were assessed, followed by induction of CFA‐induced inflammatory pain or CCI‐induced neuropathic pain and subsequent CNO administration (Figure 6A,B). Behavioral analyses showed that mechanical and thermal withdrawal thresholds were significantly reduced 1–2 days after CFA injection and 5–7 days after CCI surgery (Figure 6C,D,G,H). Chemogenetic activation of ENK^RVM→SRD^ neurons significantly elevated both mechanical and thermal withdrawal thresholds in CFA‐ and CCI‐treated mice. Notably, these antinociceptive effects returned to baseline approximately 2.5 h after CNO administration (Figure 6E,F,I,J). We further examined whether activation of the ENK^RVM→SRD^ projection induces conditioned place preference (CPP) in mice experiencing inflammatory or neuropathic pain (Figure 6K). CPP testing revealed that CFA‐ or CCI‐treated mice spent significantly more time in the CNO‐paired chamber, accompanied by increased CPP scores compared with baseline measurements in Penk‐ires‐cre::FDIO‐hM3Dq mice (Figure 6L–U). These findings indicate that activation of the ENK^RVM→SRD^ projection produces a positive motivational effect consistent with relief from ongoing pain. Collectively, these data demonstrate that activation of the ENK^RVM→SRD^ circuit effectively alleviates inflammatory and neuropathic pain hypersensitivity and is sufficient to drive pain relief–associated reward in pathological pain states.

## 4 Discussion

Our study provides the first comprehensive evidence that enkephalinergic RVM neurons, and more specifically the ENK^RVM→SRD^ projection, constitute a functionally selective descending pain‐inhibitory microcircuit. This circuit is both necessary and sufficient for gating basal nociception and for reversing inflammatory and neuropathic hypersensitivity in mice. By integrating RNAscope in situ hybridization with bidirectional chemogenetic and optogenetic manipulations, we show that ENK^RVM^ neuronal activity is robustly enhanced in CFA‐ and CCI‐induced pain states. In naïve mice, inhibition of ENK^RVM^ neurons or the ENK^RVM→SRD^ projection rapidly induces mechanical and thermal hyperalgesia, whereas activation of this pathway elevates basal pain thresholds. In contrast, in CFA‐ or CCI‐induced pain models, activation of ENK^RVM^ neurons or the ENK^RVM→SRD^ projection rapidly and reversibly alleviates established hyperalgesia. Importantly, these antinociceptive effects occur without detectable changes in locomotor function or anxiety‐like behavior, suggesting that the ENK^RVM→SRD^ pathway represents a relatively selective circuit for pain modulation.

Our results also suggest a state‐dependent dissociation between nociceptive modulation and reward‐related behavior. In naive mice, activation of ENK^RVM^ neurons elevated pain thresholds but did not induce CPP, indicating that increased antinociception alone is not sufficient to generate reward‐like behavior in the absence of ongoing pain. In contrast, in CFA‐ or CCI‐induced pain states, activation of ENK^RVM^ neurons relieved hypersensitivity and may produce relief‐associated motivation by reducing an ongoing aversive pain state. Conversely, inhibition of ENK^RVM^ signaling lowered pain thresholds and may promote aversive responses. Thus, the motivational outcome of ENK^RVM^ manipulation likely depends on whether animals are in a pain‐free or ongoing pain state.

The RVM has long been regarded as a mixed center mediating both pain facilitation (ON‐cells) and inhibition (OFF‐cells), yet the neurochemical identity and functional coupling of these populations have remained unclear [25, 26]. Using RNAscope, we found that both CFA‐ and CCI‐induced pain significantly increased the number of c‐Fos–positive ENK^RVM^ neurons, indicating that this enkephalinergic subpopulation is activated during both inflammatory and neuropathic pain states. This finding does not contradict previous reports suggesting that ENK^RVM^ neurons are predominantly inhibitory; rather, it suggests that under sustained nociceptive drive, ENK^RVM^‐mediated negative feedback is upregulated as a compensatory mechanism to counteract excessive pain transmission, although this response is insufficient to fully offset pain sensitization [27, 28, 29]. This incomplete compensation may result from persistent peripheral nociceptive input, spinal sensitization, and parallel descending facilitatory mechanisms that exceed the inhibitory capacity of ENK^RVM^‐mediated feedback. Although inhibition of pain‐activated ENK^RVM^ neurons in CFA or CCI models would further test this idea, our loss‐of‐function experiments in naive mice showed that both chemogenetic and optogenetic inhibition of ENK^RVM^ neurons rapidly lowered nociceptive thresholds, supporting the role of this population in maintaining basal antinociceptive tone.

Although our inhibition experiments indicate that ENK^RVM^ neurons and the ENK^RVM→SRD^ projection maintain basal antinociceptive tone, basal firing activity and enkephalin release were not directly measured. Because c‐Fos is a threshold‐dependent marker, low c‐Fos expression in naive mice does not exclude tonic activity. Future studies using direct recordings, transmitter‐specific labeling, peptide‐release assays, and MOR/DOR antagonists are needed to define the precise mechanism and temporal dynamics.

Projections from the RVM to downstream brainstem reticular structures, including the nucleus reticularis dorsalis (SRD), have long been considered a critical component of descending pain modulation [17, 30], However, direct causal evidence linking specific neurochemical RVM subpopulations and defined projection pathways to pain regulation has been lacking. In the present study, by combining Penk‐ires‐cre mice with a Retro‐FLEx‐FlpO and FDIO‐hM3Dq/eNpHR intersectional strategy, we achieved pathway‐ and cell‐type–specific manipulation of the ENK^RVM→SRD^ projection [31, 32]. Manipulation of this defined circuit yielded effects highly consistent with those observed following global ENK^RVM^ modulation: inhibition of the ENK^RVM→SRD^ projection reduced mechanical and thermal pain thresholds, whereas its activation elevated basal nociceptive thresholds and robustly alleviated CFA‐ and CCI‐induced hypersensitivity. Notably, selective manipulation of the ENK^RVM→SRD^ pathway did not reproduce the emotional or motor side effects often associated with broader RVM interventions or with projections to other downstream targets, such as the spinal cord. These findings indicate that the ENK^RVM→SRD^ pathway represents a functionally selective descending pain‐inhibitory circuit, preferentially modulating the sensory dimension of pain. This circuit‐level dissociation has important implications for understanding the multidimensional nature of pain processing. Our results support the concept that sensory pain modulation can be partially segregated from motor function and anxiety‐like behaviors at the level of defined brainstem microcircuits, providing a theoretical framework for the development of analgesic strategies that minimize emotional and locomotory side effects. Nevertheless, it remains likely that ENK‐negative RVM subpopulations projecting to limbic and midbrain structures, such as the amygdala and periaqueductal gray, contribute preferentially to the emotional and motivational dimensions of pain. Elucidating the organization and interaction of these parallel RVM output pathways will be an important direction for future studies [33, 34].

Chemogenetic activation of ENK^RVM^ neurons or the ENK^RVM→SRD^ pathway significantly elevated pain thresholds within 30 min, with the effects fully dissipating within approximately 2.5 h, demonstrating robust temporal controllability of this circuit. In contrast, optogenetic manipulation afforded millisecond‐level temporal precision, providing a powerful experimental framework for the future development of closed‐loop neuromodulation strategies. Given the high evolutionary conservation of enkephalinergic neurons within the RVM across rodents and humans, the ENK^RVM→SRD^ circuit may represent a promising translational target for the treatment of neuropathic and inflammatory pain.

Notably, a prior study identified a descending RVM‐to‐spinal dorsal horn pathway composed largely of GABAergic/enkephalinergic neurons, which innervates the spinal dorsal horn and bidirectionally regulates nociceptive sensitivity [15]. An important issue is how the ENK^RVM→SRD^ pathway examined here relates to previously described ENK‐expressing RVM projections to the spinal dorsal horn; the present findings should not be interpreted as excluding or replacing the established RVM‐to‐spinal dorsal horn pathway. Although several observations support a major contribution of the SRD projection to the effects observed in this study, we cannot completely exclude the possibility that sparse spinal collaterals below the detection limit, or collateral projections to other downstream sites, may contribute to some effects, particularly in chemogenetic experiments that activate SRD‐projecting cell bodies and therefore all of their axonal outputs. Future studies combining dual retrograde tracing from the SRD and spinal dorsal horn, whole‐brain and whole‐spinal‐cord axonal reconstruction, and projection‐terminal‐specific manipulations will be required to determine whether SRD‐projecting and spinally projecting ENK^RVM^ neurons are fully segregated or partially overlapping populations.

## 5 Conclusion

In sum, our findings identify ENK^RVM^ neurons and their ENK^RVM→SRD^ projection as a pivotal node within the descending pain inhibitory system. This microcircuit bidirectionally modulates nociception without engaging affective or motor components, thereby providing a refined functional framework for understanding brainstem pain control and laying a theoretical and experimental foundation for the development of precise analgesic strategies with minimal side effects.

## Author Contributions

Man Li, Xianghong Jing, and Ping Peng designed the project and drafted the manuscript; Kexing Wan and Jiajia Huang performed the experiments; Qian Xu, Chi Cui, Yulong Shi, and Jie Lei analyzed the data and interpreted the results; Zaiyan Fang, Tianyi Xu, Yufei Zheng, Jiaao Wu, and Zhengle Zhu prepared the data figures. Zhiqiang Zhang, Yujie Liang, Yichi Zhang, and Hui‐Lin Pan critically revised the manuscript. All authors approved the final version of the manuscript.

## Funding

This work was supported by the Key Program of the National Natural Science Foundation of China (grant #82130122) and Hubei Provincial Natural Science Foundation of China (grant #2025AFD807).

## Ethics Statement

All experimental procedures were approved by the Institutional Animal Care and Use Committee (IACUC) of Huazhong University of Science and Technology and carried out according to the ethical guidelines of the Helsinki Declaration of 1975 (as revised in 2008) concerning Human and Animal Rights.

## Consent

We declare that the Publisher has the author's permission to publish the relevant contribution.

## Conflicts of Interest

The authors declare no conflicts of interest.

## Supporting information


**Figure S1:** Inflammatory and neuropathic pain models induce pain hypersensitivity.
**Figure S2:** Conditioned place aversion test for Chemogenetic inhibition of ENK^RVM^ neurons.
**Figure S3:** Photoinhibition of ENK^RVM^ neurons induces hyperalgesia and real‐time place avoidance.
**Figure S4:** Chemogenetic activation of ENK^RVM^ neurons alleviates basal nociception.
**Figure S5:** Chemogenetic activation of ENK^RVM^ neuron alleviates pain hypersensitivity induced by CFA and CCI.
**Figure S6:** Quantification of the proportion of SRD‐projecting ENK^RVM^ neurons among total ENK‐positive neurons in the RVM.
**Figure S7:** Histological verification of projection specificity under the two‐virus intersectional strategy for active ENK^RVM→SRD^ circuit.
**Figure S8:** The Effects of ENK^RVM→SRD^ circuit manipulation on motor performance.

## Data Availability

The original data of the study are available from the corresponding authors with reasonable request.
