# Supplementary material for: The ENKRVM →SRD Projection Constitutes a Functionally Specific Circuit for Bidirectional Pain Modulation
Source: CNS Neurosci Ther. 2026 Jul 28;32(7):e71064. doi: 10.1002/cns.71064 (PMC13411284; doi:10.1002/cns.71064)
Supplement: Supplementary file 1 — Figure S1: Inflammatory and neuropathic pain models induce pain hypersensitivity. Figure S2: Conditioned place aversion test for Chemogenetic inhibition of ENKRVM neurons. Figure S3: Photoinhibition of ENKRVM neurons induces hyperalgesia and real‐time place avoidance. Figure S4: Chemogenetic activation of ENKRVM neurons alleviates basal nociception. Figure S5: Chemogenetic activation of ENKRVM neuron alleviates pain hypersensitivity induced by CFA and CCI. Figure S6: Quantification of the proportion of SRD‐projecting ENKRVM neurons among total ENK‐positive neurons in the RVM. Figure S7: Histological verification of projection specificity under the two‐virus intersectional strategy for active ENKRVM→SRD circuit. Figure S8: The Effects of ENKRVM→SRD circuit manipulation on motor performance. [file CNS-32-e71064-s001.docx]

Supplementary Materials for

**The ENK^RVM→SRD^ Projection Constitutes a Functionally Specific Circuit for Bidirectional Pain Modulation**

Kexing Wan et al.

* Corresponding author: Prof. Man Li

E-mail: liman73@mails.tjmu.edu.cn

Prof. Xianghong Jing

E-mail: jxhtjb@263.net

Prof. Ping Peng

E-mail: pengpingtj@163.com

1. **Supplementary Methods**

**1.1 von Frey filaments**

Mechanical sensitivity was assessed using calibrated von Frey filaments, as previously described for evaluating mechanical allodynia and paw withdrawal thresholds in rodents [1]. Mice were placed on a wire mesh platform within a transparent acrylic enclosure (22 × 10 × 14 cm) and allowed to acclimate for 30 min. After acclimation, the plantar surface of the affected hind paw was stimulated using a graded series of von Frey filaments. Each filament was applied perpendicular to the plantar surface for 3–5 s. A positive response was defined as a rapid paw withdrawal, licking, or lifting of the paw. Measurements were performed at 5‑min intervals between stimulations. The 50% mechanical withdrawal threshold was calculated using the up‑and‑down method based on six consecutive responses.

**1.2 Hot Plate Test**

Thermal nociceptive thresholds were assessed using the hot plate test, which is widely used to evaluate supraspinally organized nociceptive responses in rodents [2]. The hot plate was set at 52.5 °C. Latency was measured from placement of the mouse on the heated surface until the occurrence of a nociceptive response, defined as paw lifting, licking, or jumping. Three measurements were obtained for each mouse and averaged, with trials separated by 10‑min intervals.

**1.3 Open field test (OFT)**

The open field test (OFT) was used to assess general locomotor activity and anxiety-like behavior, consistent with previous behavioral studies using open-field parameters to evaluate locomotion and exploratory behavior in mice [3]. Mice were allowed to acclimate to the testing room for 30 min prior to testing. Animals were placed in the center of the open‑field arena (50 × 50 × 45 cm) and allowed to explore freely for 5 min. Locomotor activity and exploratory behavior were recorded and analyzed using Supermaze software (Shanghai Xinruan Information Technology Co., Ltd., Shanghai, China). The arena was cleaned with 75% ethanol between trials to eliminate olfactory cues.

**1.4 Conditioned place preference (CPP) and Conditioned place aversion (CPA)**

The CPP/CPA procedures were conducted using a three-compartment apparatus, as previously used to evaluate reward- or aversion-related motivational responses in rodents [4]. The apparatus had external dimensions of 63 × 32 × 35 cm and was separated by removable doors. The two conditioning chambers (30 × 30 × 30 cm) differed in visual and tactile cues. Chamber A featured horizontal black‑and‑white stripes and a 10 × 10 mm grid floor, whereas Chamber B featured vertical stripes and a 2 × 30 mm striped floor. Animal movement and chamber dwell time were recorded and analyzed using video‑based MOT tracking software.

The CPP/CPA protocol consisted of four phases. Acclimation (Days 1–2): Mice were allowed to explore the apparatus freely for 30 min each day. Pre‑test (Day 3): Baseline chamber preference was assessed during a 10‑min free exploration session with unrestricted access to both chambers. Conditioning (Days 4–6): Mice received intraperitoneal (i.p.) injections of saline or the treatment compound and were confined to the corresponding chamber for 30 min. For chemogenetic experiments, clozapine‑N‑oxide (CNO; 3.0 mg/kg) was administered, and mice were confined to Chamber B for 30 min beginning 0.5–1 h after injection; the CNO session was conducted 4 h after the saline session. Post‑test (Day 7): Mice were allowed to freely explore both chambers for 10 min. CPP/CPA scores were calculated as the difference in time spent (seconds) between the test chamber (Chamber B/CNO) and the control chamber (Chamber A/saline).

**1.5 Real-Time Place Aversion (RTPA)**

The real-time place avoidance (RTPA) assay was performed with reference to previous optogenetic studies using real-time place aversion to evaluate negative motivational effects induced by neural circuit manipulation [5]. The RTPA apparatus consisted of two chambers (30 × 30 × 30 cm each) connected by a 10 × 30 cm doorway. Chamber A featured vertical black‑and‑white stripes and a striped floor, whereas Chamber B featured horizontal white stripes and a perforated circular floor. Animal movement and chamber dwell time were recorded and analyzed using Video MOT software.The RTPA protocol consisted of four phases. Acclimation (Days 1–2): Mice were allowed to explore both chambers freely for 30 min each day. Pre‑test: Mice explored both chambers for 10 min in the absence of optical stimulation to determine baseline chamber preference. Stimulation phase: Mice were allowed to explore the apparatus freely for 10 min. Upon entry into the preferred chamber, ENK‑positive neurons were optogenetically stimulated with yellow light (589 nm, 1 Hz, 999‑ms pulse width, 10 mW). Optical stimulation was terminated when the mouse exited the preferred chamber and entered the non‑preferred chamber. Post‑test: Mice were allowed to explore both chambers freely for 10 min without optical stimulation.

**1.6 Real-Time Place Preference (RTPP)**

The real-time place preference (RTPP) assay was performed with reference to previous optogenetic RTPP paradigms used to assess positive motivational effects of neural circuit activation [6]. The RTPP setup was identical to that used for the RTPA experiment. The protocol was similar, except that blue light stimulation (473 nm, 20 Hz, 5‑ms pulse width, ~5 mW) was delivered when the mouse entered or remained in the non‑preferred chamber. The stimulation phase lasted 10 min, followed by a 10‑min post‑test consisting of free exploration without optical stimulation. Animal movement and chamber dwell time were recorded and analyzed using Video MOT software.

1. **Supplementary Figures**


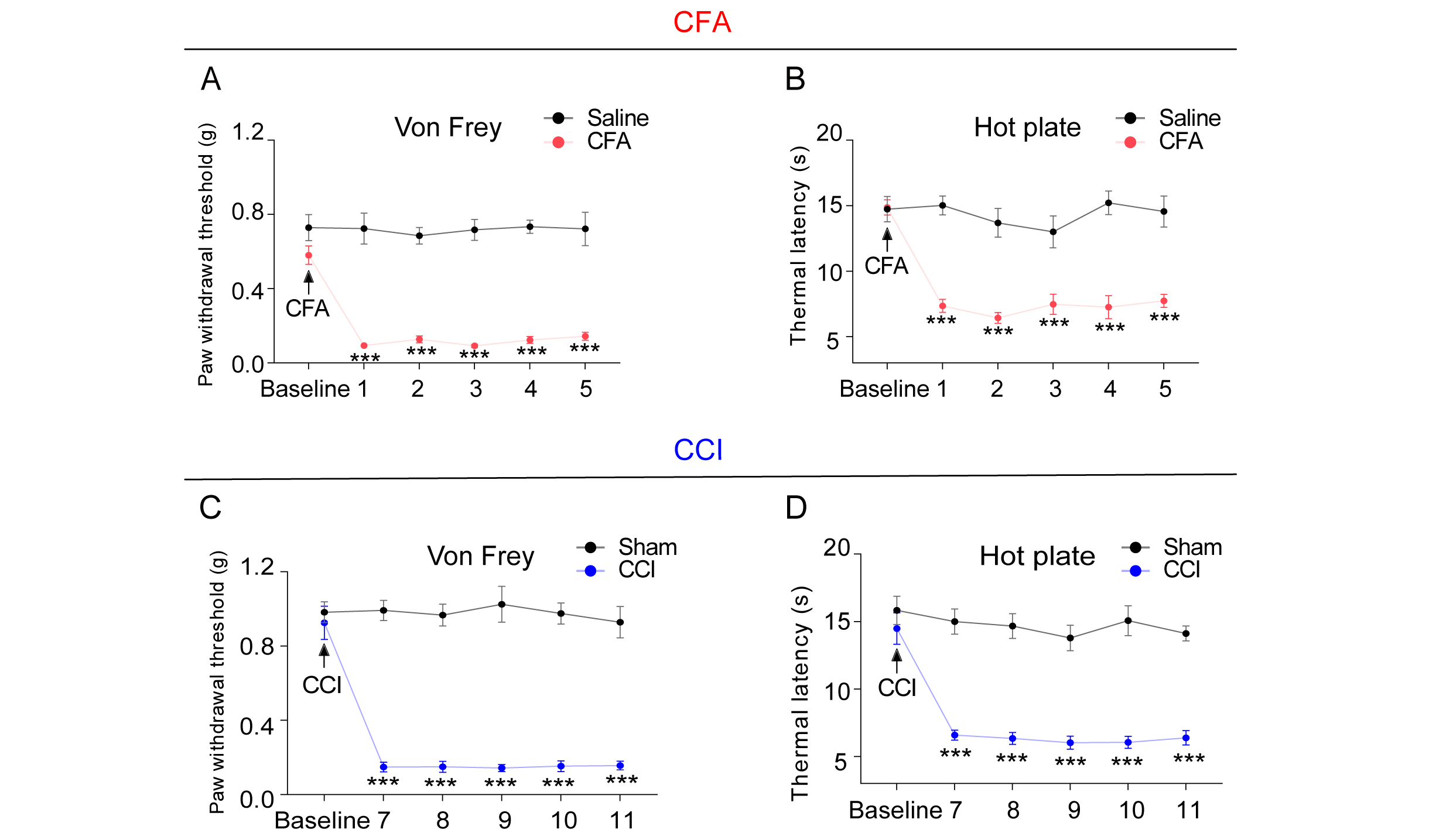


**Supplementary Fig. 1 Inflammatory and neuropathic pain models induce pain hypersensitivity.**

1. Time course of tactile withdrawal thresholds measured by von Frey testing in the CFA model. (B) Time course of thermal withdrawal latency measured by the hot plate test in the CFA model. (C) Time course of tactile withdrawal thresholds measured by von Frey testing in the CCI model. (D) Time course of thermal withdrawal latency measured by the hot plate test in the CCI model. ****P* < 0.001 vs. saline (CFA) or sham (CCI), two‑way ANOVA with Bonferroni’s post hoc test; n = 6 mice per group.


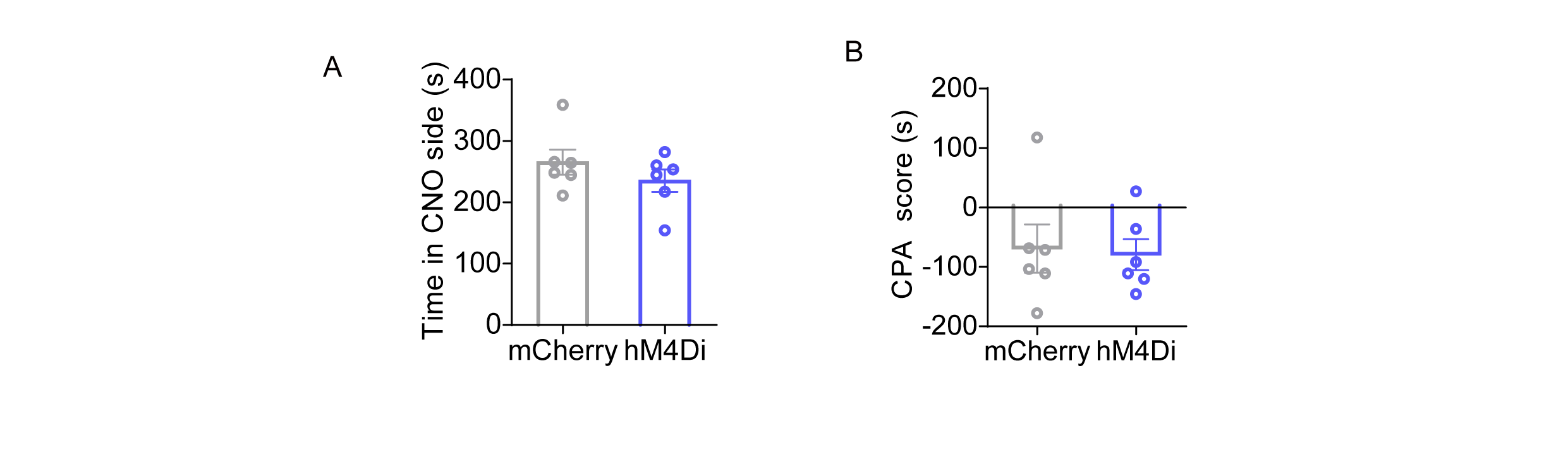


**Supplementary Fig. 2 Conditioned place aversion test for Chemogenetic inhibition of ENK^RVM^ neurons.**

(A, B) Time spent in the CNO-paired chamber (A) and CPA score (B) in Penk-cre::DIO-mCherry control mice and Penk-cre::DIO-hM4Di mice.


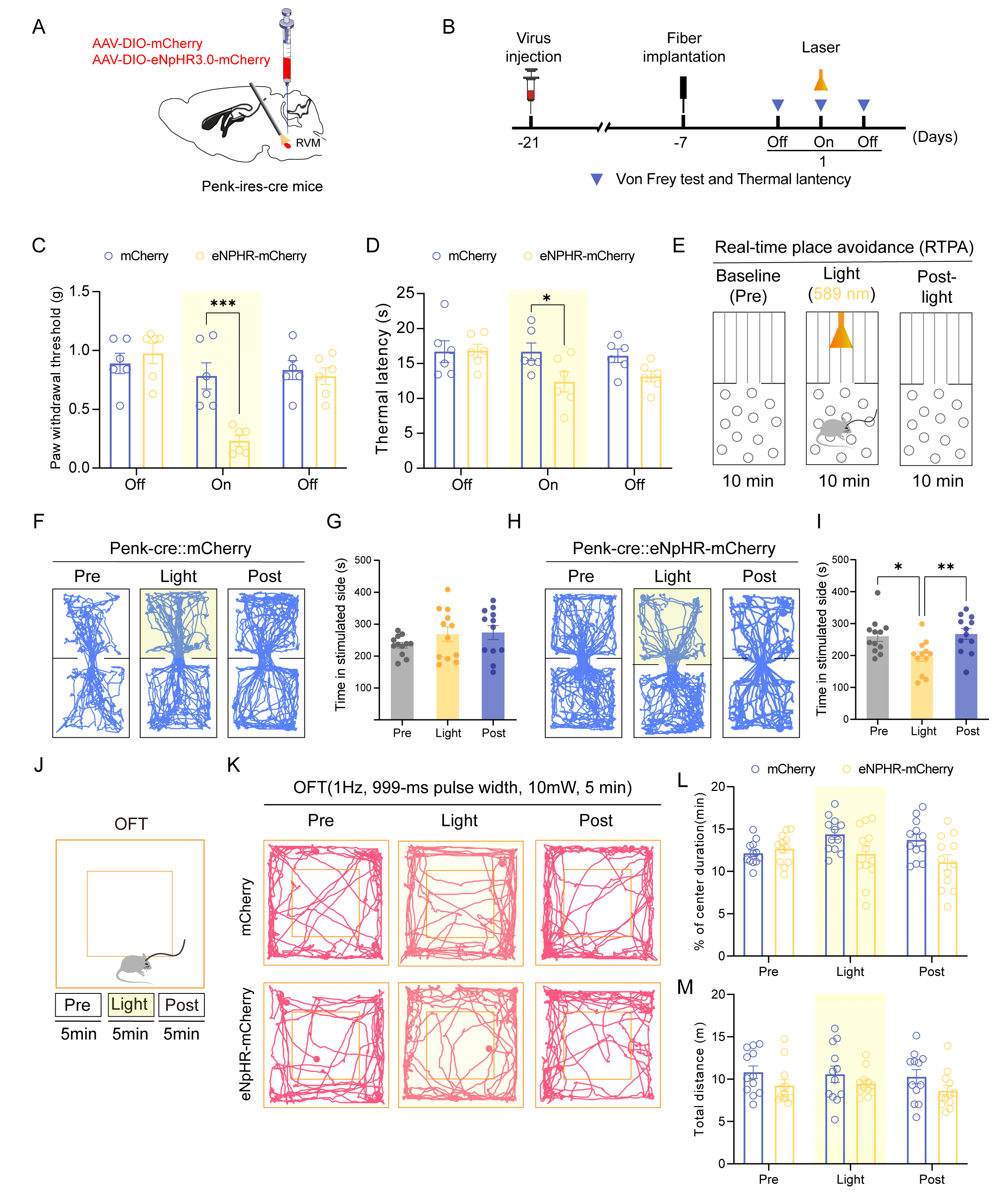


**Supplementary Fig. 3 Photoinhibition of ENK^RVM^ neurons induces hyperalgesia and real‑time place avoidance.**

(A) Diagram illustrating the stereotaxic injection strategy for expression of AAV‑DIO‑eNpHR3.0‑mCherry or AAV‑DIO‑mCherry in the RVM of Penk‑ires‑Cre mice. (B) Schematic of the optical illumination strategy. (C) Yellow light illumination (589 nm, 1 Hz, 999‑ms pulse width, 10 mW) of the RVM significantly decreased the paw withdrawal threshold (PWT) in response to mechanical stimulation in mice expressing DIO‑eNpHR3.0‑mCherry, but not in mice expressing AAV‑DIO‑mCherry. (D) Yellow light illumination (589 nm, 1 Hz, 999‑ms pulse width, 10 mW) of the RVM significantly decreased the latency of the thermal paw‑withdrawal response in the hot plate test in mice expressing DIO‑eNpHR3.0‑mCherry, but not in mice expressing AAV‑DIO‑mCherry. (E) Schematic of the real‑time place avoidance (RTPA) test. (F) Representative RTPA tracking paths illustrating behavioral responses during yellow (589 nm) light illumination in mice injected with AAV‑DIO‑mCherry in the RVM. (G) Quantification of RTPA performance before (Pre), during (Light), and after (Post) 10‑min yellow light illumination of the RVM in mice injected with AAV‑DIO‑mCherry. (H) Representative RTPA tracking paths illustrating behavioral responses during yellow (589 nm) light illumination in mice injected with DIO‑eNpHR3.0‑mCherry in the RVM. (I) Quantification of RTPA performance before (Pre), during (Light), and after (Post) 10‑min yellow light illumination of the RVM in mice injected with DIO‑eNpHR3.0‑mCherry. (J) Schematic of the open‑field test (OFT) conducted before (Pre), during (Light), and after (Post) 5‑min photoinhibition (589 nm, 1 Hz, 999‑ms pulse width, 10 mW) of ENK^RVM^ neurons. (K) Representative OFT tracking paths from Penk‑ires‑Cre mice expressing AAV‑DIO‑mCherry (upper) or DIO‑eNpHR3.0‑mCherry (lower). (L–M) Quantification of total distance traveled (L) and the ratio of time spent in the periphery and center (M) during the OFT before (Pre), during (Light), and after (Post) 5‑min yellow light illumination of the RVM in mice expressing DIO‑eNpHR3.0‑mCherry or AAV‑DIO‑mCherry.All data are presented as mean ± SEM. Error bars represent SEM. **P* < 0.05, **P < 0.01.


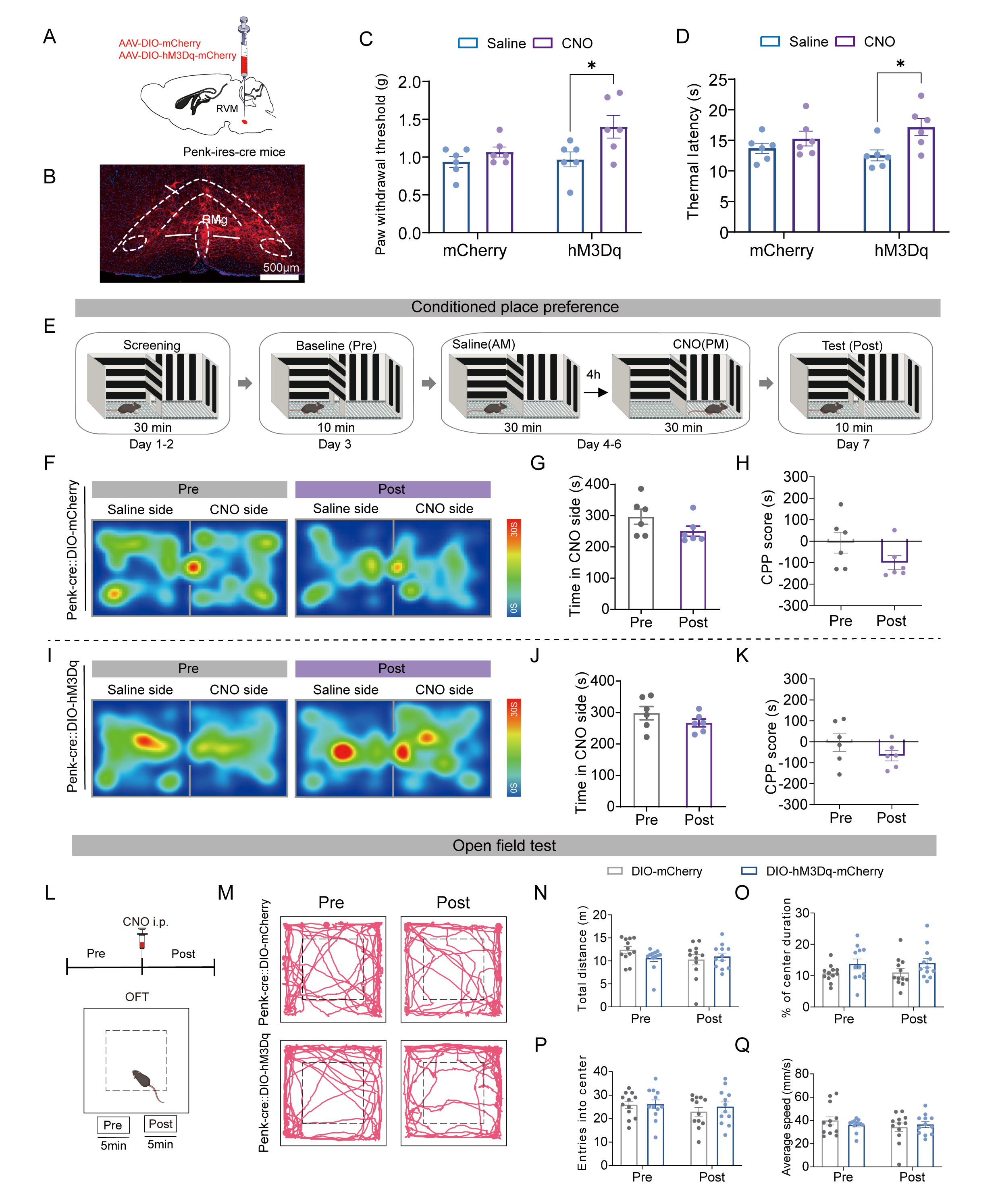


**Supplementary Fig. 4** **Chemogenetic** **activation of ENK^RVM^ neurons** **alleviates basal nociception.**

(A) Diagram illustrating stereotaxic injection of AAV‑DIO‑hM3Dq‑mCherry or AAV‑DIO‑mCherry into the RVM of Penk‑ires‑Cre mice. (B) Representative fluorescence image showing the viral injection site. Scale bar, 500 μm. (C) Effect of chemogenetic activation of ENK^RVM^ neurons on tactile withdrawal thresholds. (D) Effect of chemogenetic activation of ENK^RVM^ neurons on thermal withdrawal latency (n = 6). For (C–D), **P* < 0.05 vs. saline treatment within the mCherry or hM3Dq group, two‑way ANOVA followed by Bonferroni’s post hoc tests. (E) Schematic of the experimental design for the conditioned place preference (CPP) test. (F) Representative CPP tracking paths from Penk‑Cre mice expressing DIO‑mCherry before and after saline and CNO conditioning. (G–H) Time spent in the CNO‑conditioned chamber (G) and CPP score (H) in Penk‑Cre mice expressing DIO‑mCherry. (I) Representative CPP tracking paths from Penk‑Cre mice expressing DIO‑hM3Dq before and after saline and CNO conditioning. (J–K) Time spent in the CNO‑conditioned chamber (J) and CPP score (K) in Penk‑Cre mice expressing DIO‑hM3Dq (n = 6). (L) Schematic of the experimental design for the open‑field test (OFT). (M) Representative OFT tracking paths from Penk‑Cre mice expressing DIO‑mCherry or DIO‑hM3Dq before and after CNO injection. (N–Q) Effects of chemogenetic activation of ENK^RVM^ neurons on total distance traveled (N), the ratio of time spent in the center (O), center entries (P), and average speed (Q) in the open field (n = 12 mice per group). All data are presented as mean ± SEM.


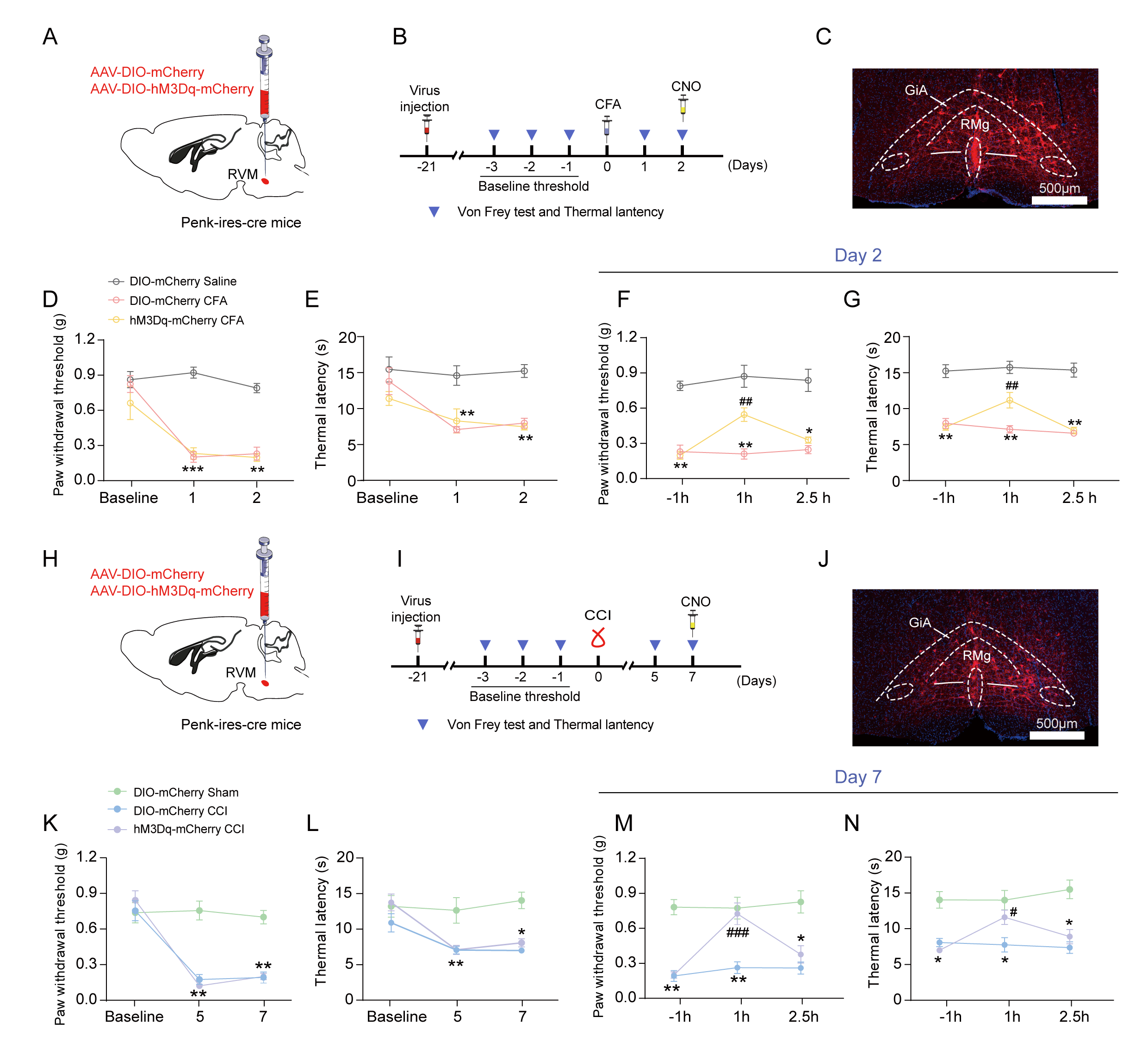


**Supplementary Fig. 5 Chemogenetic activation of ENK^RVM^ neuron alleviates pain hypersensitivity induced by CFA and CCI.**

(A) Diagram illustrating stereotaxic injection of AAV‑DIO‑hM3Dq‑mCherry or AAV‑DIO‑mCherry into the RVM of Penk‑ires‑Cre mice. (B) Experimental design and timeline of behavioral testing for chemogenetic activation of ENK^RVM^ neurons in the CFA model. (C) Representative fluorescence image showing the viral injection site. Scale bar, 500 μm. (D-E) Time course of paw withdrawal thresholds (D) and thermal withdrawal latency (E) in Penk‑Cre mice expressing DIO‑mCherry or DIO‑hM3Dq following CFA treatment. (F-G) Time course of paw withdrawal thresholds (F) and thermal withdrawal latency (G) in CFA‑treated Penk‑ires‑Cre mice expressing DIO‑mCherry or DIO‑hM3Dq after CNO administration. (H) Diagram illustrating stereotaxic injection of AAV‑DIO‑hM3Dq‑mCherry or AAV‑DIO‑mCherry into the RVM of Penk‑ires‑Cre mice. (I) Experimental design and timeline of behavioral testing for chemogenetic activation of ENK^RVM^ neurons in the CCI model. (J) Representative fluorescence image showing the viral injection site. Scale bar, 500 μm. (K-L) Time course of paw withdrawal thresholds (K) and thermal withdrawal latency (L) in Penk‑ires‑Cre mice expressing DIO‑mCherry or DIO‑hM3Dq following CCI surgery. (M-N) Time course of paw withdrawal thresholds (M) and thermal withdrawal latency (N) in CCI‑treated Penk‑ires‑Cre mice expressing DIO‑mCherry or DIO‑hM3Dq after CNO administration. **P* < 0.05, ***P* < 0.01, ****P* < 0.001 vs. saline (CFA) or sham (CCI) control groups; #*P* < 0.05, ##*P* < 0.01, ###*P* < 0.001 vs. CFA‑ or CCI‑treated DIO‑mCherry groups; two‑way ANOVA followed by Bonferroni’s post hoc tests; n = 6 mice per group. All data are presented as mean ± SEM.


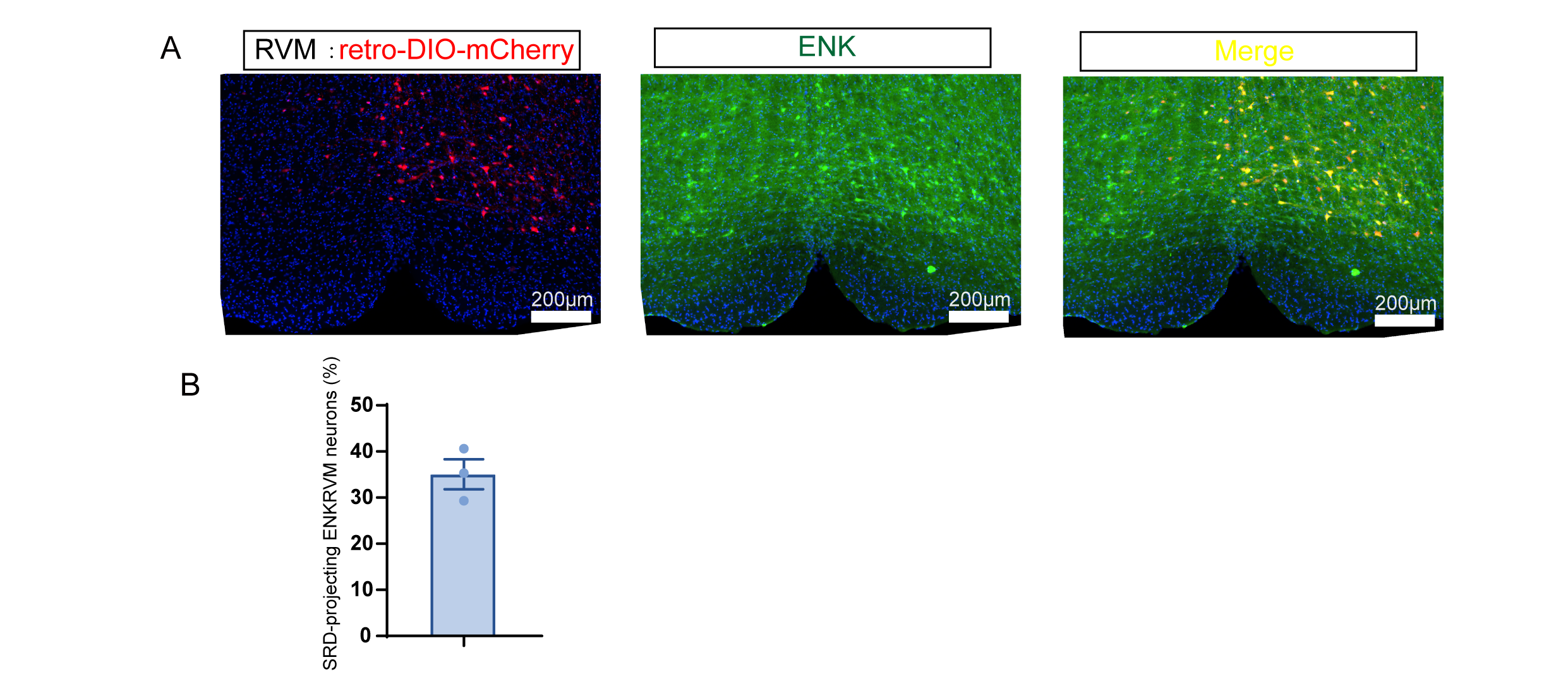


**Supplementary Fig. 6 Quantification of the proportion of SRD-projecting ENK^RVM^ neurons among total ENK-positive neurons in the RVM.**

1. IF staining for SRD-projecting ENK^RVM^ neurons among total ENK-positive neurons in the RVM. (B) Quantification of the proportion of SRD-projecting ENK^RVM^ neurons among total ENK-positive neurons in the RVM, n=3. Scale bars = 200 μm.


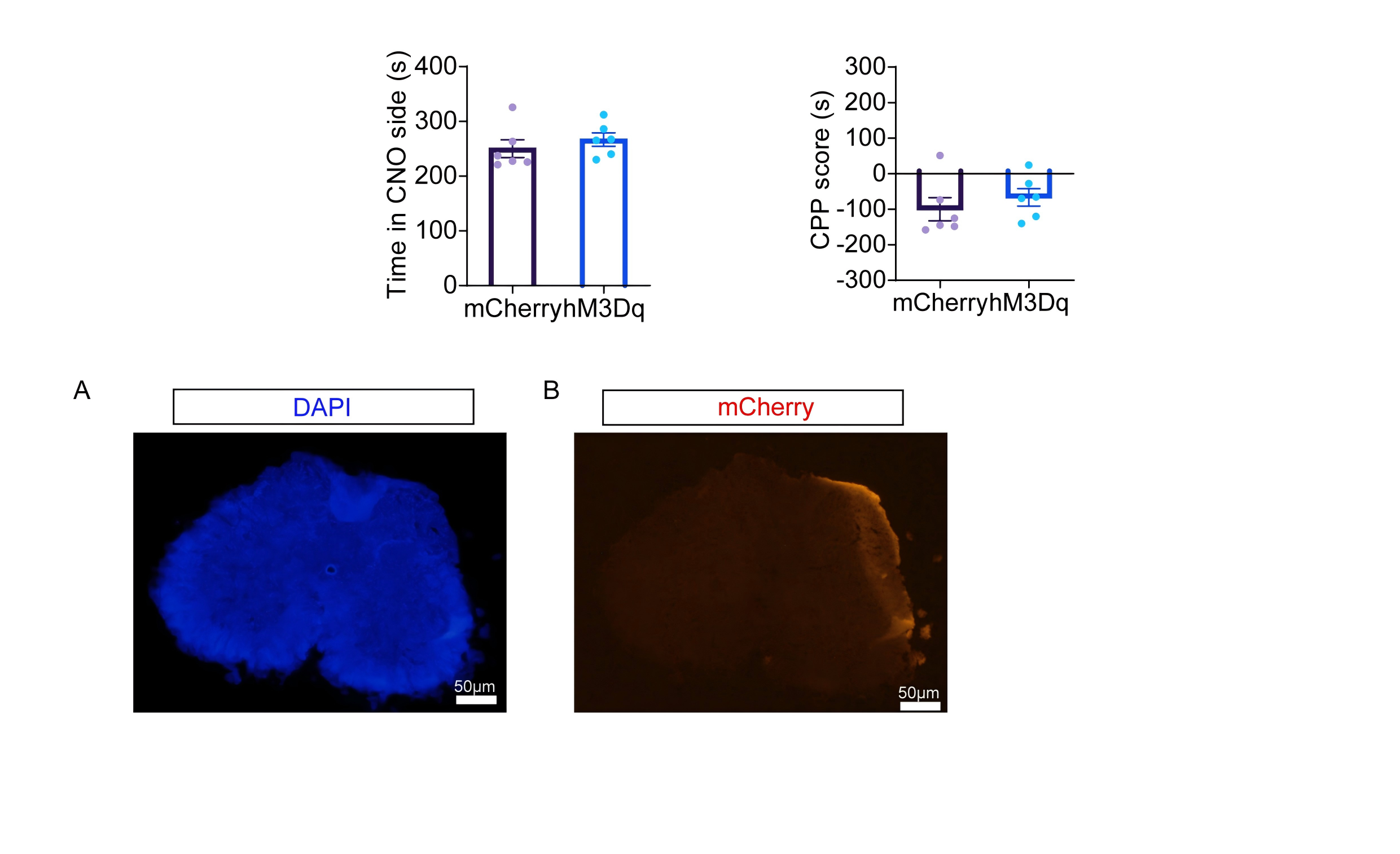


**Supplementary Fig. 7 Histological verification of projection specificity under the two-virus intersectional strategy for active ENK^RVM→SRD^ circuit.**

AAVretro-DIO-FLEx-FlpO was injected into the SRD, together with the AAV-FDIO-hM3Dq-mCherry viral strategy in the RVM of Penk-ires-cre mice, (A, B) Representative spinal cord sections showing no detectable mCherry-positive labeled somata or axonal projections in the spinal dorsal horn. Scale bars = 50 μm.


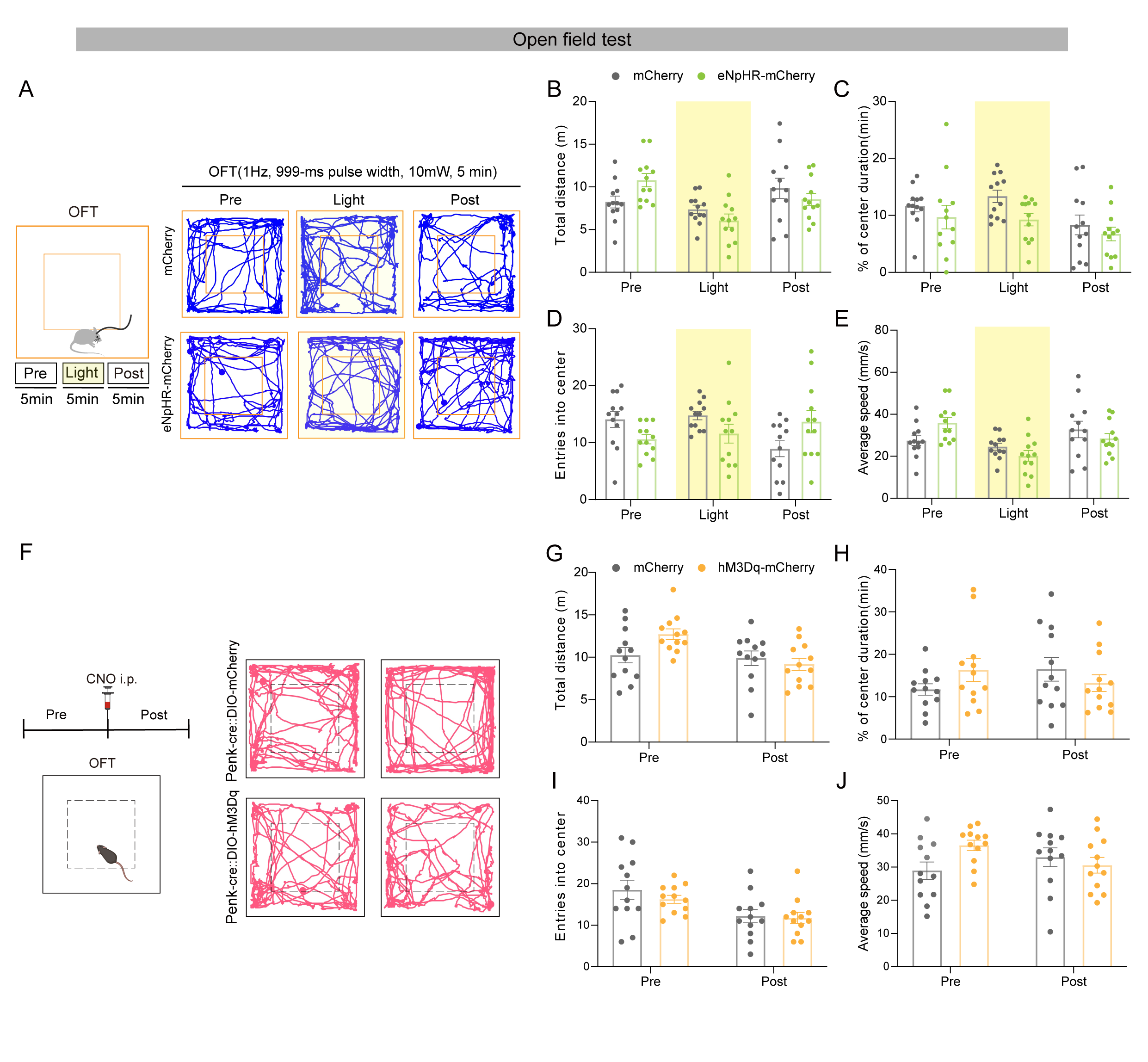


**Supplementary Fig. 8 Effects of** **ENK^RVM→SRD^ circuit manipulation on motor performance.**

1. Schematic illustration of the open field test (OFT) performed before (Pre), during (Light), and after (Post) 5 min of photoinhibition (589 nm, 1 Hz, 999-ms pulse width, 10 mW) of ENK^RVM→SRD^ neurons (left), along with representative locomotor trajectories of Penk-ires-cre mice transfected with AAV-DIO-mCherry (upper) or AAV-DIO-eNpHR3.0-mCherry (lower). (B–E) Quantification of total distance traveled (B), percentage of time spent in the center (C), number of center entries (D), and mean velocity (E) in the OFT before (Pre), during (Light), and after (Post) 5 min of yellow light illumination of the SRD in mice transfected with AAV-DIO-eNpHR3.0-mCherry or AAV-DIO-mCherry in RVM. (F) Representative locomotor tracking maps of Penk-ires-cre::DIO-mCherry and Penk-ires-cre::DIO-hM3Dq mice in the OFT before and after clozapine-N-oxide (CNO) administration.(G–J) Effects of chemogenetic activation of ENK^RVM→SRD^ neurons on total distance traveled (G), percentage of time spent in the center (H), number of center entries (I), and mean velocity (J) in the OFT (n = 12 mice per group).All data are presented as mean ± SEM.

**Reference** (citation number in the Supplementary Materials text)

1. Modi AD, Parekh A, Pancholi YN. Evaluating pain behaviours: Widely used mechanical and thermal methods in rodents. Behav Brain Res. 2023 May 28;446:114417. doi: 10.1016/j.bbr.2023.114417.
2. Gunn A, Bobeck EN, Weber C, Morgan MM. The influence of non-nociceptive factors on hot-plate latency in rats. J Pain. 2011 Feb;12(2):222-7. doi: 10.1016/j.jpain.2010.06.011.
3. Paterno R, Marafiga JR, Ramsay H, Li T, Salvati KA, Baraban SC. Hippocampal gamma and sharp-wave ripple oscillations are altered in a Cntnap2 mouse model of autism spectrum disorder. Cell Rep. 2021 Nov 9;37(6):109970. doi: 10.1016/j.celrep.2021.109970.
4. Davoody L, Quiton RL, Lucas JM, Ji Y, Keller A, Masri R. Conditioned place preference reveals tonic pain in an animal model of central pain. J Pain. 2011 Aug;12(8):868-74. doi: 10.1016/j.jpain.2011.01.010.
5. Siemian JN, Arenivar MA, Sarsfield S, Borja CB, Russell CN, Aponte Y. Lateral hypothalamic LEPR neurons drive appetitive but not consummatory behaviors. Cell Rep. 2021 Aug 24;36(8):109615. doi: 10.1016/j.celrep.2021.109615.
6. Siemian JN, Arenivar MA, Sarsfield S, Aponte Y. Hypothalamic control of interoceptive hunger. Curr Biol. 2021 Sep 13;31(17):3797-3809.e5. doi: 10.1016/j.cub.2021.06.048.
